# Morphological dormancy, embryo growth and pericarp restraint during crop and wild Apiaceae mericarp germination in response to ambient temperature

**DOI:** 10.1007/s00425-025-04850-7

**Published:** 2025-11-01

**Authors:** Kazumi Nakabayashi, Lena M. M. Fatelnig, Matthew Walker, Sue Kennedy, James E. Hourston, Ondřej Novák, Danuše Tarkowská, Miroslav Strnad, Frances Gawthrop, Tina Steinbrecher, Gerhard Leubner-Metzger

**Affiliations:** 1 Department of Biological Sciences, Royal Holloway University of London, Egham, Surrey TW20 0EX UK; 2 Department of Agro-Environmental Science, Obihiro University of Agriculture and Veterinary Medicine, Obihiro, Hokkaido 080-8555 Japan; 3 Tozer Seeds Ltd, Cobham, Surrey KT11 3EH UK; 4 Elsoms Seeds Ltd, Spalding, Lincolnshire PE11 1QG UK; 5 Eden Research Plc, Milton Park, Oxfordshire, OX14 4SA UK; 6 Laboratory of Growth Regulators, Institute of Experimental Botany, Palacký University Olomouc, Czech Academy of Sciences and Faculty of Science, 77900 Olomouc, Czech Republic

**Keywords:** *Apium graveolens* (celery), Chilling stress, *Daucus carota* (carrot), Embryo growth, Morphological dormancy, *Pastinaca sativa* (parsnip), Pericarp (fruit coat) biomechanics, Thermal-time modelling, Thermoinhibition, Wild Apiaceae species

## Abstract

**Main conclusion:**

Apiaceae morphological dormancy and germination differ between crop and wild species, and among crop cultivars in the mechanical, hormonal and thermal mechanisms that control pericarp (fruit coat) weakening and pre-gremination embryo growth.

**Abstract:**

The Apiaceae disperse morphologically (MD) or morphophysiologically dormant mericarps, indehicent fruits in which the single seed is encased by the pericarp (fruit coat) and the underdeveloped (small) embryo is embedded in abundant living endosperm tissue. Pre-germination embryo growth from an initial to a critical relative embryo size (embryo:fruit or embryo:seed length ratio) is a requirement for the completion of germination by radicle emergence. The roles and mechanisms of pre-gremination embryo growth and pericarp constraint were investigated by embryo-growth imaging, pericarp ablation/biomechanics, tissue-specific hormone analytics, and population-based thermal-time threshold modelling. Comparison of Apiaceae crop cultivars, including *Pastinaca sativa* (parsnip), *Apium graveolens* (celery) and *Daucus carota* (carrot) with > 50 wild Apiaceae species revealed that the initial relative embryo sizes of crop species are significantly larger compared to wild species. Interestingly, the critical relative embryo sizes of the phylogenetic group that contains parsnip, were smaller for the crop compared to wild species. ABA-insensitive and auxin-promoted pre-germination embryo growth was blocked by heat (thermoinhibition), while the completion of germination by radicle emergence was inhibited by ABA. The thick pericarp of parsnip decreased in thickness and mechanical tissue resistance in parallel with the pre-germination embryo growth, while the thin pericarps of celery and carrot did not change. Parsnip pericarp contained significantly higher contents of the germination-inhibiting hormones abscisic acid (ABA) and *cis*-(+)-12-oxo-phytodienoic acid (*cis*-OPDA) compared to celery pericarp. Pericarp ablation experiments revealed that it acts as a mechanical and chemical (ABA, *cis*-OPDA) constraint (coat component of MD), and has a key role in narrowing the permissive temperature window for germination.

**Supplementary Information:**

The online version contains supplementary material available at 10.1007/s00425-025-04850-7.

## Introduction

Germination timing of plant fruits and seeds is achieved through a diversity of dormancy mechanisms that provide adaptation to the prevailing environment, ensuring that the chances for successful seedling establishment are maximised (Baskin and Baskin 2014; Finch-Savage and Footitt 2017). The Apiaceae (carrot family) comprises many horticultural crops, such as carrot, celery, parsnip, fennel, dill and parsley, which are known for their germination problems in horticultural practice (Robinson 1954). The Apiaceae produce dry schizocarps as fruits, which split into individual mericarps upon maturity. These mericarps are the dispersal and germination units, and they constitute dry single-seeded indehiscent fruit halves in which a dead outer pericarp (fruit coat) is encasing the seed (Liu et al. 2006, 2012; Wojewodzka et al. 2019). These publications highlight the taxonomic and phylogenetic value of the Apiaceae morphological fruit diversity, as well as the importance of pericarp morphological diversity for mericarp dispersal strategies. In species of the Brassicaceae and Amaranthaceae with dry indehiscent fruits it is known that the pericarp may confer a mechanical or chemical constraint to the completion of germination (Sperber et al. 2017; Mohammed et al. 2019; Hourston et al. 2022; Chandler et al. 2024; Steinbrecher et al. 2025). Biomechanical pericarp constraint, e.g. by blocking or delaying water uptake, is known for *Raphanus* spp. and *Lepidium didymum* (Sperber et al. 2017; Steinbrecher et al. 2025). Biochemical pericarp constraint, e.g. by storing and leaching germination inhibitors includes phenolic compounds (Ignatz et al. 2019) and plant hormones such as abscisic acid (ABA) (Mohammed et al. 2019). The mechanisms by which the Apiaceae pericarp may control mericarp germination, whether pericarps differ between Apiaceae species, and what role(s) they play in germination responses to nonoptimal temperatures are largely unknown.

The internal morphology of mature seeds differs in their relative embryo size (embyo:seed or embryo:fruit length ratio), which represents a key trait in the seed morphospace (Baskin and Baskin 2014; Carta et al. 2024). Relative embryo size has shaped angiosperm history at major evolutionary and climatic events, but its adaptive significance, ecological role and contribution to seed dormancy are largely unknown. A hallmark of Apiaceae internal seed structure is that they have morphological (MD) or morphophysiological (MPD) dormancy (Homrichhausen et al. 2003; Baskin and Baskin 2014; Walker et al. 2021). MD and MPD seeds are characterised by containing underdeveloped (small) embryos embedded in abundant endosperm tissue of mature seeds, which was proposed to be the ancestral state in seed dormancy evolution (Willis et al. 2014). These underdeveloped embryos must first grow inside the imbibed MD/MPD seeds to reach a critical embryo:seed (E:S) or embryo:fruit (E:F) length ratio before germination can be completed by radicle emergence (Jacobsen et al. 1976; Jacobsen and Pressman 1979; Homrichhausen et al. 2003; Walck and Hidayati 2004; Scholten et al. 2009; Vandelook et al. 2012; Baskin and Baskin 2014; Porceddu et al. 2017; Zhang et al. 2019a; Walker et al. 2021, 2025; Visscher et al. 2022; Blandino et al. 2025). The physiological component of this dormancy is usually lost in horticultural Apiaceae crops, including *Daucus carota* (carrot) (Homrichhausen et al. 2003; Nascimento et al. 2013) and *Apium graveolens* (celery) (Biddington and Thomas 1978; Walker et al. 2021, 2025), and these species consequently have MD. Ecophysiological studies suggest that there is a very close association between MD and MPD with numerous species capable of exhibiting both kinds of dormancy, as well as multiple levels of MPD, within a single species, population, or plant (Baskin and Baskin 1979, 1990b, 2021; Walck et al. 2008; Hawkins et al. 2010; Zardari et al. 2019).

Distinct hormone-temperature interactions and molecular mechanisms underpin the reduced embryo growth in response to sub-optimal and supra-optimal temperatures. In earlier work (Walker et al. 2021, 2025) with MD celery mericarps, we demonstrated that hormone-temperature interactions affect the pre-germination embryo growth inside imbibed celery mericarps and the associated endosperm degradation to reach a critical relative embryo size required for radicle emergence. The regulation of these processes is controlled by a complex interaction between gibberellin (GA), auxin (IAA, indole-3-acetic acid), and abscisic acid (ABA) metabolism and changes in the tissue-specific sensitivities to these hormones. In contrast to this, during chilling and across the entire sub-optimal temperature range of celery (6–20 °C), the initiation of embryo growth was delayed in a thermal-time compliant manner, as was the expression of GA-induced genes encoding endo-*β*−1,4-mannanase and expansin important for ABA-insenitive endosperm degradation and embryo growth. Moreover, a biphasic nature of endosperm breakdown became apparent, with the main bulk of endosperm breakdown being less ABA-sensitive and associated with resource mobilisation to facilitate cavity formation and to fuel embryo growth (phase II). In contrast to this, the late stage (phase III) is strongly ABA-sensitive and controls radicle protrusion probably by restraint weakening of the micropylar endosperm and pericarp as seen in non-dormant and physiologically dormant seeds (Yan et al. 2014; Chahtane et al. 2017; Steinbrecher and Leubner-Metzger 2017). The embryo growth and endosperm degradation inside imbibed celery mericarps (Walker et al. 2021, 2025) is not simply the completion of embryogenesis or *Arabidopsis thaliana* equivalent post-embryogenesis growth, but a distinct process as revealed by the hormonal mechanisms, embryo-endosperm interactions and the spatiotemporal expression patterns of corresponding genes.

Here, we compare horticultural Apiaceae crop cultivars (celery, carrot, parsnip, others) with > 50 wild Apiaceae species and identified group-specific differences between crop and wild species in their initial and critical relative embryo sizes. Crop and wild Apiaceae species also differed in their temperature requirements and thermal ranges for mericarp germination, as well as in their pericarp (fruit coat) thickness. This raised the intriguing question if pericarp restraint plays a role in mericarp germination and thermal responses. To investigate this, we compared parsnip (thick pericarp) with celery (thin pericarp) mericarp germination by thermal-time modelling, embryo growth and hormone analysis, and by pericarp biomechanics and ablation experiments. Pericarp ablation enhanced parsnip germination and widened its permissive temperature window. The pericarp was identified as a key compartment that determines germination timing. It is proposed that higher contents of the germination inhibitors abscisic acid (ABA) and *cis*-(+)−12-oxo-phytodienoic acid (*cis*-OPDA) in the parsnip pericarp is, at least in part, a mechanism that enhances dormancy and narrows the permissive temperature window for germination.

## Materials and methods

### Plant material

Fruits of Apiaceae are dry schizocarps that break into two dispersal units, which are single-seeded mericarps. In the current work, we use the term ‘fruit’ to refer to the mericarp. Fruits of the *Apium graveolens* L. (celery) F1 hybrid cultivars Victoria, Monterey and Loretta (lot no. 03731094, 03731245, 037245382, Suplementary Table S1) were harvested in 2014, kept in company warehouse storage at 14 °C with a relative humidity of 25% sealed in foil bags, and provided for the current research work by Tozer Seeds Ltd. (Cobham, Surrey, UK) as described by Walker et al. (2021). Fruits of *Daucus carota* L. (carrot) cultivar Nerac (lot no. E56490 (2018), graded 1.6–1.8 mm) and *Pastinaca sativa* L. (parsnip) F1 hybrid cultivars Panorama (#1 lot no. E64291 (2019), #2 lot no. E57306 (2018), #3 lot no. E38814/E38817) and Pacific (#1 lot no. E64292 (2019), #2 lot no. E57305 (2018), Suplementary Table S1) were provided by Elsoms Seeds Ltd. (Spalding, Lincolnshire, UK). Parsnip fruit lots #1 and #3 were pre-washed and for #1 the 4.75–5.00 mm caliber fraction was used; for #3 the germination results at different temperatures for the 4.75–5.00 mm (E38814) and 3.75–4.00 mm (E38817) calibers did not differ. Fruit aliquots for experimental use were stored at 4 °C (Panorama #3) or 20 °C (Nerac, Panorama #1 and #2, Pacific #1 and #2) in airtight containers containing silica for ~ 6–12 months prior to conducting the experiments.

### Germination assays

Germination assays were performed in a Panasonic MLR-352 Environmental Test Chamber (Panasonic, Bracknell, UK) set to 14 °C, 20 °C, 24 °C, or 32 °C as indicated, and continuous white light (~ 100 μmol s^−1^ m^−2^). Temperature response assays for the thermal-time modelling were performed on a GRD1-LH temperature gradient plate device (Grant Instruments Ltd., Cambridge, UK). Triplicates of 50 (celery, carrot) or 30 (parsnip) fruits were used per treatment, with each triplicate sown into a 6-cm-diameter Petri dish with two filter papers (MN713, Macherey–Nagel, Dueren, Germany) and 2 ml of autoclaved deionised water. For comparative experiments of parsnip cultivar Panorama #3 between fruits and seeds, true seeds were obtained by manually removing the fruit coats (pericarps). Germination was defined as the visible emergence of the radicle through all the encasing tissues. Dose–response germination assays were performed using gibberellin A_4+7_ (GA_4+7_; Duchefa Biochemie, Haarlem, The Netherlands), *cis*,t*rans*-S+)-abscisic acid (ABA, Duchefa), fluridone (Duchefa), or diphenyl methylphosphonate (DMP, Sigma-Aldrich, St. Louis, MO, USA) at the indicated concentrations. These hormones or inhibitors were added to the germination assays from concentrated stock solutions with either water or, for GA_4+7_, dimethyl sulfoxide (DMSO) as solvent. The germination curves of water and DMSO controls did not differ (Walker et al. 2021). All treatments contained 0.1% plant preservative mixture PPM (Plant Cell Technology, Washington, USA). Where indicated, diphenyl methylphosponate (DMP) was used as a triacyl glycerol inhibitor (Brown et al. 2013).

### Embryo growth assays and imaging

Internal embryo growth within imbibed fruits or seeds was assessed at the temperatures and times indicated using the same incubation conditions as the germination assays. Approximately 50 fruits were used per time point, to measure sizes they were longitudinally cut and photographed using a Leica DCF480 digital camera attached to a Leica Mz 12,5 stereomicroscope (Leica, Wetzlar, Germany). The embryo and fruit lengths were measured via the image analysis software ImageJ v1.6.0 (National Institute of Health, Bethesda, MD, USA). Embryo (E) sizes were represented as the ratio of the seed (S) length (E:S ratio) or the ratio of the fruit/mericarp (F) length (E:F ratio) to account for the embryo-seed/fruit size association. Germinated fruits or seeds were removed, and their values were replaced by a mean critical E:S or E:F ratio for radicle protrusion. The critical E:S or E:F ratio was calculated by measuring the internal embryo, seed and fruit lengths of 50 fruits or seeds where the radicle had just protruded, as described in detail by Walker et al. (2021). Pericarp thickness during imbibition was calculated from the difference in fruit and seed length.

### Biomechanical measurements

Puncture force (PF) measurements were conducted as described previously using a modified custom-made biomechanics device (Steinbrecher and Leubner-Metzger 2017; Steinbrecher et al. 2025). In brief, a rounded metal pin with 0.5 mm diameter was driven into the parsnip sample at 0.7 mm/min while force and displacement were recorded simultaneously. For measurement, parsnip fruits or seeds (pericarp manually removed) were halved and placed in a 3D-printed sample holder with a cavity in the shape of a semi-ellipse. Parsnips were cut in half (split along the minor axis) and the half not containing the embryo (distant-half endosperm) was measured. Pericarp PF during imbibition was calculated from the difference in fruit and seed PF.

### Plant hormone extraction and quantification

For whole fruit quantification of gibberellins, abscisates, jasmonates and auxins, Petri-dishes of fruits were prepared as described for the germination assays. Five replicates were used for each time point, with approximately 100 fruits used per replicate sample. In addition to whole fruit quantification, at the 1, 3 and 5-day (celery) and 7-day (parsnip) time points' fruits were also separated into their three core compartments (endosperm, embryo and testa + pericarp) for tissue-specific hormone quantification as described in detail by Walker et al. (2021). The levels of gibberellin (GA) metabolites, ABA, phaseic acid (PA), dihydrophaseic acid (DPA), indole-3-acetic acid (IAA), jasmonic acid (JA), jasmonoyl-L-isoleucine (JA-Ile) and *cis*-(+)−12-oxo-phytodienoic acid (*cis*-OPDA) were quantified by UHPLC-MS/MS as described (Urbanova et al. 2013; Flokova et al. 2014; Simura et al. 2018; Walker et al. 2021; Chandler et al. 2024).

### Population-based thermal-time threshold modelling and statistical analysis

The cardinal temperatures permissible for germination, including base temperature (*T*_b_), optimal temperature (*T*_o_) and ceiling (maximal) temperature (*T*_c_), and the thermal time constants Θ were identified by population-based threshold modelling for thermal time (Finch-Savage and Leubner-Metzger 2006; Batlla and Benech-Arnold 2015; Bradford and Bello 2022) as described in detail in our earlier work (Loades et al. 2023; Steinbrecher et al. 2025; Walker et al. 2025). In brief, germination rates GR_g%_, i.e. the inverse of time to germination for a given percentage of the population (1/*t*_g%_), were plotted against the imbibition temperatures. Best goodness of fit (*R*^2^) lines in the sub-optimal (colder) and supra-optimal (warmer) temperature regions were calculated by linear regression analysis with the GraphPad Prism v10 (GraphPad Software Inc., San Diego, CA, USA) programme. *T*_b_ and *T*_c_ were estimated from their intercepts with the *x*-axis. The estimated *T*_b_ fitting all fractions was used to recalculate regression lines forced through this *T*_b_ value for the linear lines in the sub-optimal temperature range. The estimated *T*_c(50%)_ and equal slopes were used for the linear lines in the supra-optimal temperature range. Linear lines in the suboptimal and supra-optimal temperature ranges were used to estimate *T*_o_ from the intersections of the regression lines. The thermal time constants (Θ_cold(g)_ and Θ_warm(g)_) were derived as the inverse of their slopes. At sub-optimal temperatures, Θ_cold(g)_ is normally distributed within the population around Θ_cold(50%)_. At supra-optimal temperatures, *T*_c(g)_ is normally distributed around *T*_c(50%)_. All statistical analyses were performed in GraphPad Prism v10 (GraphPad Software Inc.).

## Results

### Relative embryo size and growth differs in crop and wild Apiaceae species

**Fig. 1:**
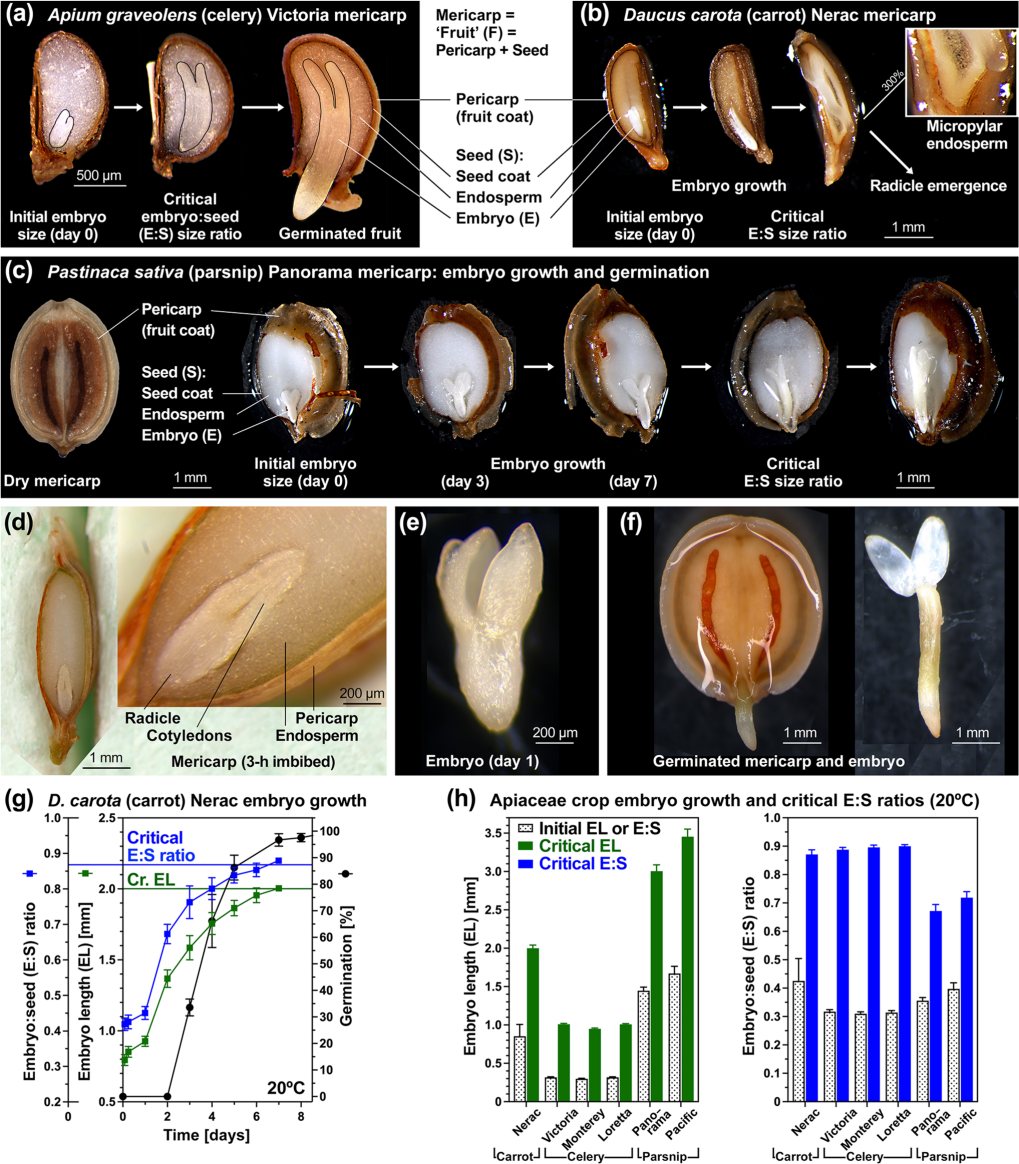
Comparative morphology and embryo growth analysis inside Apiaceae mericarps. **a** Microscopic images of *Apium graveolens* cultivar Victoria mericarps (hereafter termed fruits) show that the morphological dormancy (MD) requires that the small embryo grows within the fruit. This occurs from the start of imbibition at 20 °C in continuous white light onwards to its critical size required for the completion of germination by radicle emergence through the micropylar endosperm, seed coat and fruit coat (pericarp); modified from Walker et al. (2021). **b** Microscopic images of *Daucus carota* cultivar Nerac MD fruits and embryo growth during imbibition. **c**–**f** Microscopic images of *Pastinaca sativa* cultivar Panorama MD fruits and embryo growth during imbibition. **g** Kinetics of germination and embryo growth from initial to critical embryo lenghts (EL) and embryo:seed size ratios (E:S) inside imbibed carrot fruits cultivar Nerac at 20 °C in continuous white light. **h** Initial and critical embryo lengths (EL, *left panel*) and embryo:seed size ratios (E:S, *right panel*) of carrot, celery and parsnip cultivars inside fruits imbibed at 20 °C in continuous white light. Mean ± SE, EL and E:S values are presented for 50 embryos (embryo and seed (excluding pericarp) size were measured); mean ± SE germination values presented are from triplicate plates each with 50 carrot fruits

**Fig. 2:**
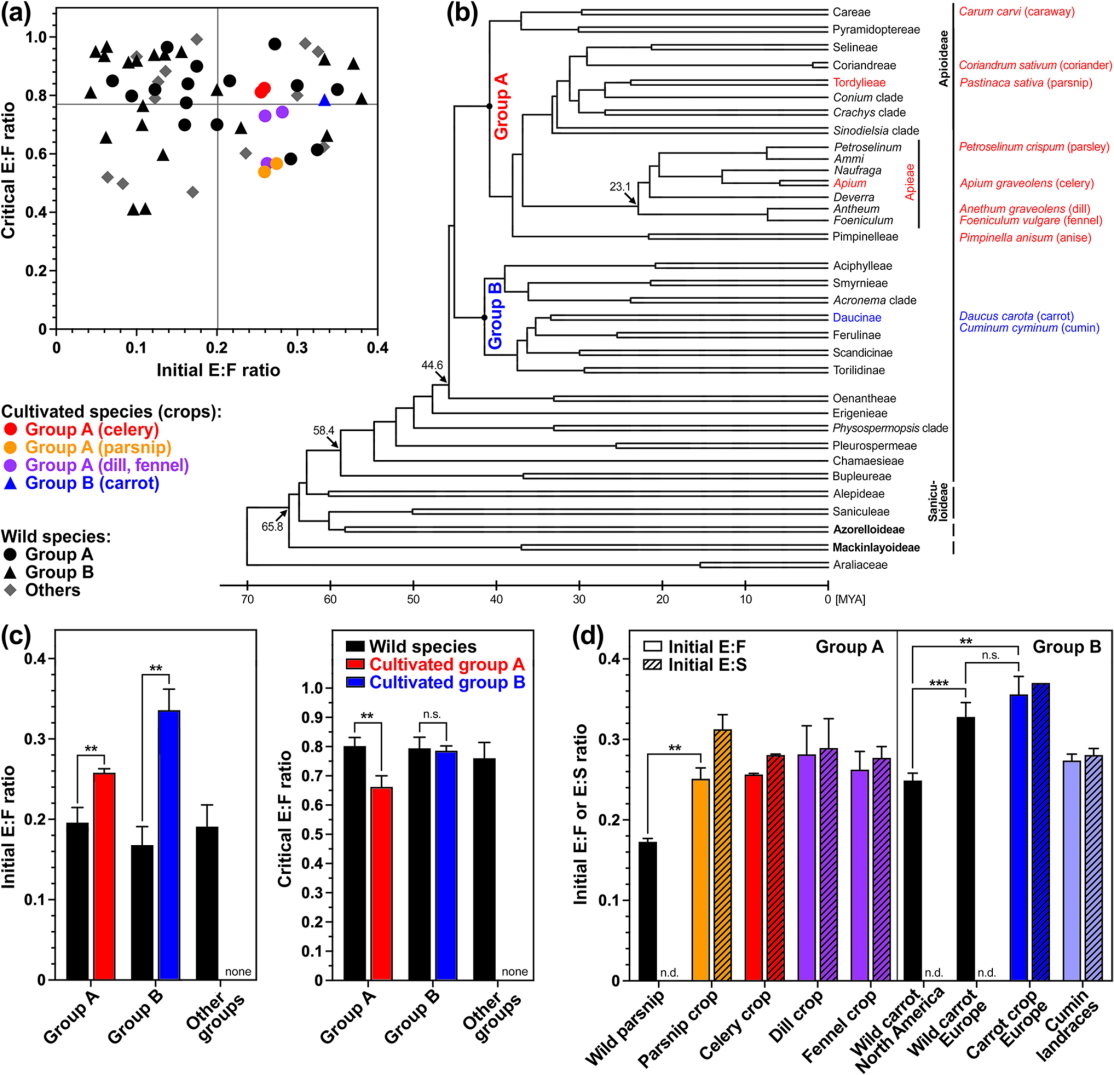
Comparison and evolution of relative embryo sizes in cultivated and wild Apiaceae species. **a** Comparison of initial and critical embryo:fruit (E:F) ratios of 7 cultivated (crop cultivars) and > 50 wild Apiaceae (Suppl. Table S2) of distinct phylogenetic groups. The gey lines indicate the mean initial (0.200) and critical (0.765) E:F ratios of all Apiaceae species (Suppl. Table S2). **b** Simplified phylogenetic tree of the Apiaceae and examples of crop species for the phylogenetic groups A and B; crop species are not known for the other Apiaceae groups. Phylogeny modified from Walker et al. (2021). **c** Mean ± SE values of initial and critical E:F ratios of wild and cultivated Apiaceae phylogenetic groups. **d** Mean ± SE values of initial and critical E:F and E:S (embryo:seed) ratios of Apiaceae crops and their wild relatives. Statistical significance of comparisons indicated was obtained using Welch’s unpaired *t* test; *P* values *** < 0.001, ** < 0.01, * < 0.05. See Suppl. Table S2 for the dataset with values, details and literature for the results presented

*Apium graveolens* (celery, Fig. 1a), *Daucus carota* (carrot, Fig. 1b), and *Pastinaca sativa* (parsnip, Fig. 1c–f) fruits differ considerably in their masses and sizes (Supplementary Table S1), but their common internal seed structures with small (underdeveloped) embryos embedded in abundant living endosperm tissue (Fig. 1) and their phylogenetic relatedness (Fig. 2) suggests common germination mechanisms. In earlier work (Walker et al. 2021) we demonstrated that the morphological dormancy (MD) of the Victoria celery cultivar requires that the small embryo must first grow inside the imbibed fruit from an initial embryo-seed (E:S) length ratio of 0.32 ± 0.01 to the critical E:S length ratio of 0.89 ± 0.01 prior to the completion of germination by radicle emergence; very similar critical E:S ratios were required for other celery cultivars (Fig. 1h).

The ~ twofold larger fruits of the carrot cultivar Nerac also required a very similar critical E:S ratio of 0.87 ± 0.02 for the completion of germination (Fig. 1b,g,h). Although celery, carrot and parsnip cultivars differ considerably in mass and size (Supplementary Table S1), their relative initial embryo sizes (E:S ratios) were very similar ~ 0.35 (Fig. 1h). The ~ fivefold larger fruits (compared to celery) of the parsnip cultivars Panorama (Fig. 1c–f) and Pacific required critical E:S ratios between 0.66 and 0.73 for the completion of germination (Fig. 1h). Parsnip embryos were fully differentiated in radicle and cotyledons already in dry fruits and also visible during early imbibition (Fig. 1c–f). As for celery and carrot (Kim and Janick 1991; de Miranda et al. 2017; Walker et al. 2021), embryo growth inside imbibed parsnip fruits at 20 °C (Fig. 1b) was associated with endosperm degradation and fueled, as for celery and carrot, by mobilisation of the stored lipids (Supplementary Fig. S1). Other Apiaceae crop species, including *Foeniculum vulgare* (fennel, Hashemirad et al. 2023) and *Anethum graveolens* (dill, Soldatenko et al. 2020; Bukharov et al. 2021) were very similar in their initial and critical relative embryo sizes (Fig. 2a, Supplementary Table S2).

Most Apiaceae crops, including parsnip, celery, dill and fennel, belong to group A, while carrot belongs to group B in the Apiaceae phylogenetic tree (Fig. 2b). To investigate if relative embryo size as a morphological fruit (mericarp) component differs between cultivated and wild Apiaceae fruits we compared their initial and critical embryo:fruit (E:F) ratios (Fig. 2, Supplementary Table S2). For this, a dataset with initial and critical E:F ratios for > 50 wild Apiaceae species was compiled (Supplementary Table S2) from published work (including Hendrix et al. 1991; Vandelook et al. 2007a, b, 2024; Scholten et al. 2009; Fasih and Afshari 2018; Blandino et al. 2019; Kadluczka and Grzebelus 2022; Visscher et al. 2022; Zhang et al. 2023; Rahimi et al. 2024; for further references see Supplementary Table S2). The initial E:F ratios of all cultivated Apiaceae species were high compared to the wild Apiaceae species, well above the wild species initial E:F mean value (0.200), while the critical E:F values of cultivated and wild species was scattered around the mean (0.765) of all Apiaceae species (Fig. 2a). Interestingly, comparison of initial E:F mean values revealed that for group A and group B, the initial E:F ratios of cultivated species were significantly larger compared to wild species (Fig. 1c). Further to this, comparison of critical E:F mean values revealed that for group A the critical E:F ratios of cultivated species were significantly smaller as compared to wild species (Fig. 1c). In contrast to this, the critical E:F mean values of group B cultivated and wild species did not differ.

For parsnip (group A) the initial E:F ratios of wild parsnip (Hendrix et al. 1991) were significantly lower compared to cultivated parsnip (Fig. 2d), and the initial E:F ratios of other group A crops were very similar (Fig. 2d). For carrot (group B), Vandelook et al. (2024) found in their work on the functional germination traits of wild carrot that the initial E:F ratios of European accessions were significantly larger compared to those of North American accessions (Fig. 2d). Interestingly, also the initial E:F ratios of European carrot cultivars were larger (Fig. 2d). The initial E:F ratios of Iranian and Indian *Cuminum cyminum* (cumin) landraces (Soltani et al. 2019) were 0.274 above the group B average for (0.187, mean of cultivated plus wild species), but smaller when compared to carrot crop cultivars (Supplementary Table S2). These cumin landraces also had MPD (Soltani et al. 2019) as most of the wild species, while most of the cultivated Apiaceae species had MD. Taken together, wild and cultivated Apiaceae species seem to differ regarding their initial E:F ratios (increased in crops), group A also for their critical E:F ratios (reduced in crops), as well as in dormancy class (MPD dominant in wild species, MD in crops), which suggests that breeding may have affected these germination traits.

### Biomechanical mechanisms of pericarp constraint in Apiaceae fruit germination

**Fig. 3:**
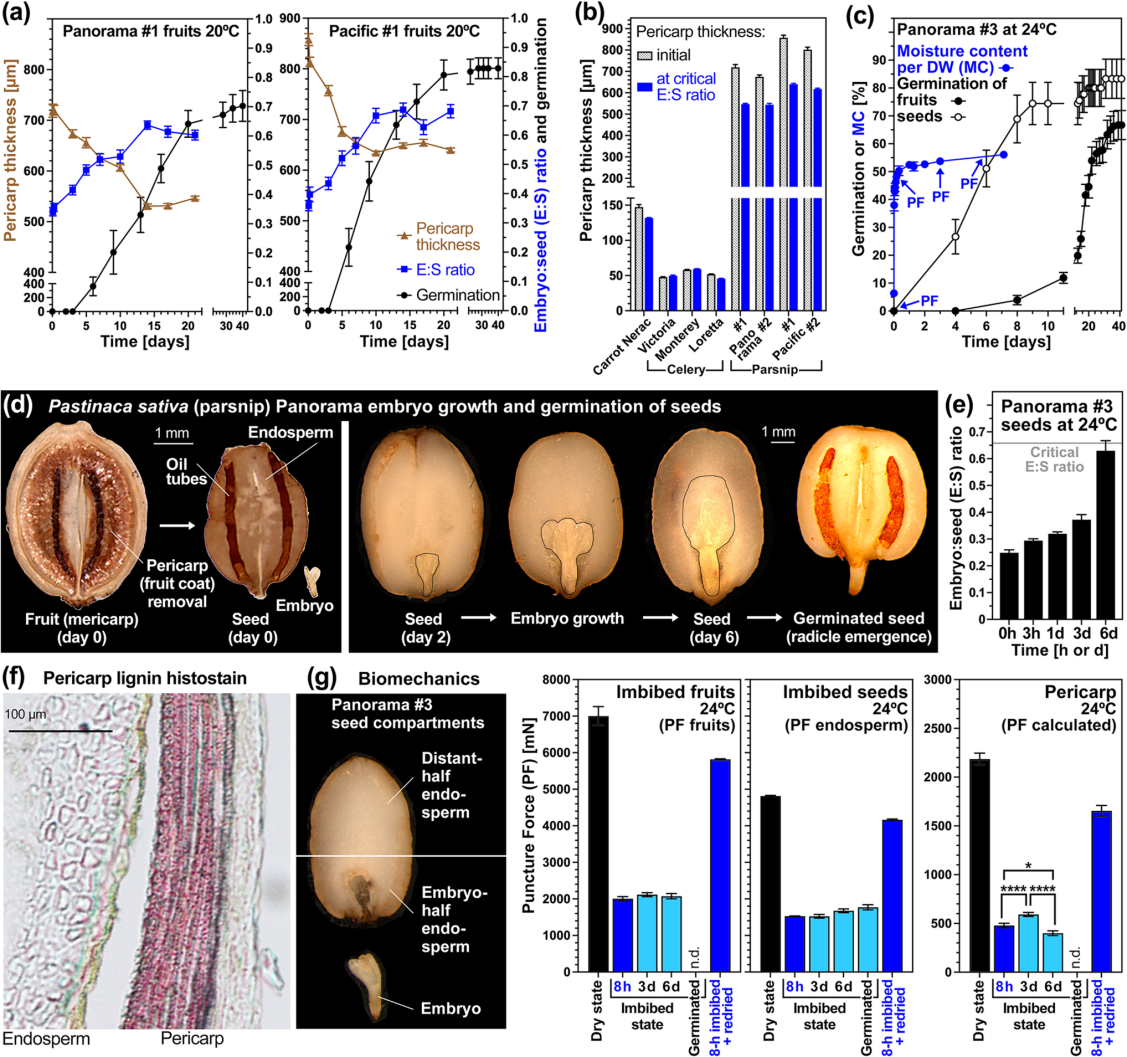
The roles of the pericarp as a germination constraint and its biomechanical properties in imbibed parsnip fruits. **a** Pericarp thickness, embryo growth, as embry:seed (E:S) length ratio, and germination of parsnip Panorama #1 and Pacific #2 fruits. Mean ± SE values for pericarp thickness and embryo length are presented from 50 imbibed fruits. Mean ± SE values for germination at 20 °C in continues white light are from triplicate plates each with 30 parsnip fruits. **b** Comparison of initial pericarp thickness and pericarp thickness at the critical E:S ratio of carrot, celery and parsnip cultivars and batches. See Supplementary Fig. S2 for detail pericarp thickness kinetics during imbibition. **c** Kinetics of parsnip seed versus fruit germination and water uptake, i.e. moisture content (MC) per dry weight (DW), during imbibition at 24 °C in continues white light. Arrows indicate the times at which puncture force (PF) was quantified. **d** Morphological analysis of embryo growth inside imbibed parsnip seeds of the Panorama #3 batch. Parsnip seeds were obtained by manually removing the pericarp in the dry state and imbibing the obtained seeds at 24 °C in continuous white light. **e** Kinetics of embryo:seed (E:S) length ratio of imbibed parsnip Panorama #3 seeds. The critical E:S ratio is indicated and reached faster (~ 6 days) in seeds as compared to fruits (~ 2–3 weeks). **f** Lignin histostaining (phloroglucinol) of parsnip Panorama #3 fruit seection showing the endosperm and pericarp. Note that the endocarp layer stains heavily for lignin. **g** Biomechanical analysis of parsnip Panorama #3 fruits and seeds during imbibition. Puncture force (PF) measurements of the distal half of imbibed fruits and seeds were conducted over time. The PF values for the pericarp were derived by substracting the seed PF values from the fruit PF values. Note that imbibition caused a three–fivefold reduction in PF for all the endosperm and the pericarp, and that re-drying reverted this to a large part. Mean ± SE values are presented obtained from 30 fruits or seeds. Statistical significance of comparisons indicated was obtained using Welch’s unpaired *t* test; *P* values **** < 0.0001, * < 0.05

Distinct differences in critical E:S and E:F values (Fig. 2d) already suggested that pericarp (fruit coat) thickness differed considerably between Apiaceae species. With ~ 50 µm and ~ 150 µm the pericarps of celery and carrot were thin compared to the thick pericarp of parsnip (~ 750 µm). Interestingly, the parsnip pericarp decreased in thickness ~ 1.3-fold during fruit imbibition in parallel with the pre-germination embryo growth (Fig. 3a, b). In contrast to parsnip, the thin celery and carrot pericarps only decreased ~ 1.1-fold during fruit imbibition (Fig. 3b, Supplemental Fig. S2). To test how the pericarp affects pre-germination embryo growth and germination we compared parsnip fruits with true seeds (Fig. 3c–e). Obtaining parsnip (true) seeds was by pericarp removal, i.e. the pericarp was manually peeled off in the dry state using Panorama batch #3 (Fig. 3d). Subsequently, seeds (without pericarp) and fruits (seeds with pericarp) were comparatively analysed for embryo growth and germination (Fig. 3c). Embryo growth inside parsnip seeds reached the critical E:S ratio within 6 days (Fig. 3e) and > 70% of the seeds completed germination within 8 days (Fig. 3c). Compared to this, embryo growth inside fruits and germination of intact fruits was ~ threefold slower (Fig. 3a). We conclude that the parsnip pericarp is a germination contraint which together with pre-germination embryo growth determines germination timing.

Pericarp removal of celery also promoted germination and increased its uniformity (Supplementary Fig. S3). This promotion was associated with the micropylar pericarp, which in intact fruits ruptures just prior to celery micropylar endosperm rupture and radicle protrusion (Supplementary Fig. S3). The parsnip pericarp contains an endocarp consisting of lignified dead tissue layers (Fig. 3f). To analyse if the endosperm and the pericarp biomechanically weaken during imbibition, we compared the puncture force (PF) of dry and imbibed parsnip fruits and seeds (Fig. 3g). Compared to the dry state, the tissue resistance (PF values) of imbibed distal-half endosperm and pericarp were ~ 3.2-fold and ~ 4.5-fold lower, respectively; they increased ~ threefold by redrying. The distal-half endosperm PF values remained roughly unchanged during imbibition. In contrast to this, the pericarp tissue resistance was significantly decreased on day 6 when compared to day 3 and 8 h (Fig. 3g). This decrease in pericarp tissue resistance was associated with the observed decrease in pericarp thickness (Fig. 3a, Supplementary Fig. S3). We therefore conclude that there is mechanical tissue weakening in the parsnip pericarp associated with pre-germination embryo growth and radicle emergence.

### Control of parsnip fruit germination by pericarp-associated hormonal inhibitors

**Fig. 4:**
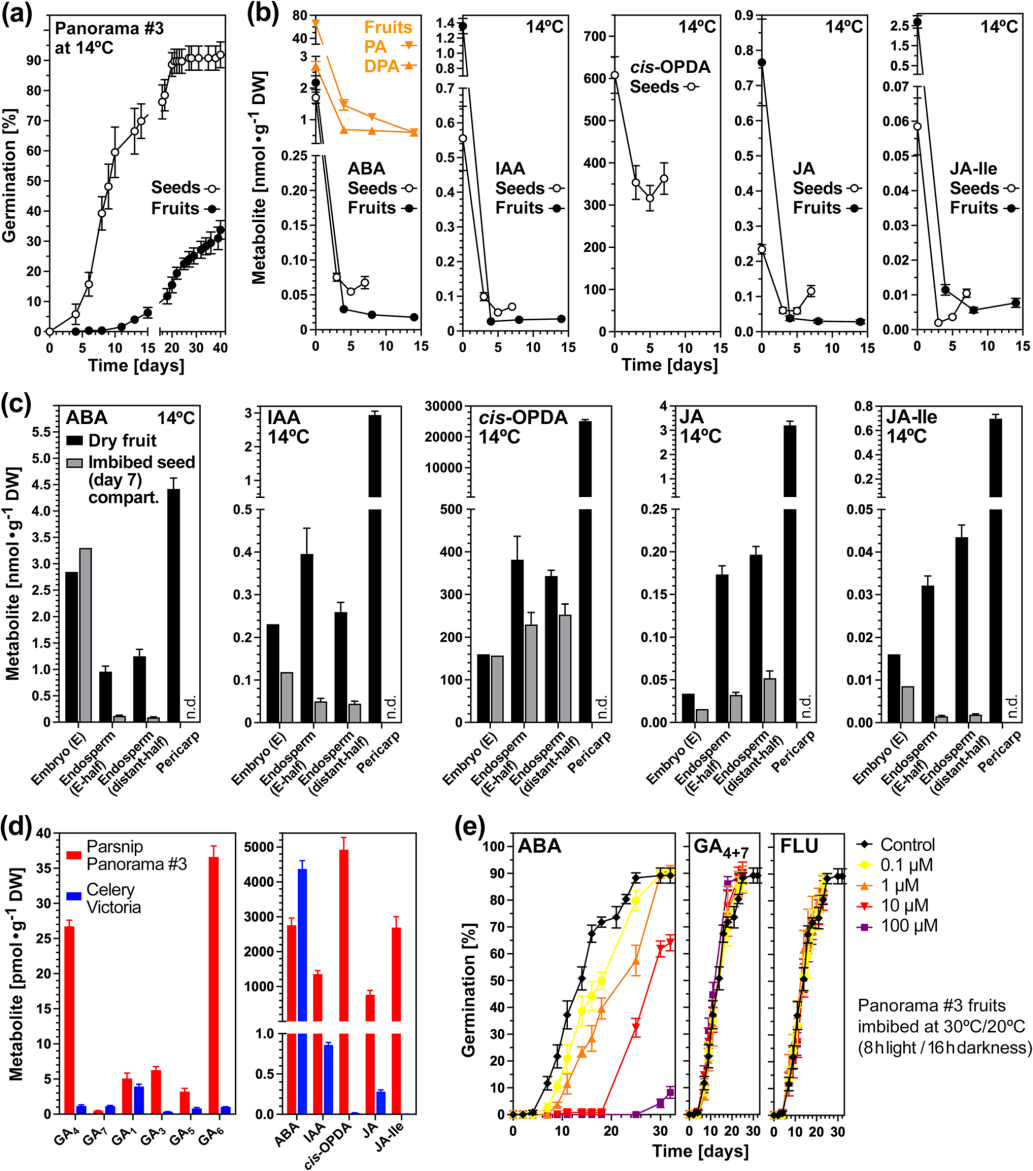
Analysis of hormone contents in fruits, (true) seeds and fruit compartments of parsnip Panorama #3. **a** Kinetics of parsnip seed versus fruit germination during imbibition at 14 °C in continues white light. Mean ± SE values are presented from triplicate plates each with 30 parsnip fruits. **b** Hormonal analysis of parsnip fruits versus seeds over time. The contents of absicic acid (ABA) and its degradation products phaseic acid (PA) and dihydrophaseic acid (DPA), and of indole-3-acetic acid (IAA), *cis*-(+)−12-oxo-phytodienoic acid (*cis*-OPDA), jasmonic acid (JA), and jasmonoyl-L-isoleucine (JA-Ile) were quantified. Mean ± SE values are presented obtained from five biological replicates with ~ 100 fruits used per replicate sample. **c** Hormonal analysis of parsnip fruit compartments embryo, endosperm-halves, and pericarp in the dry state and after 7 days of imbibition at 14 °C. **d** Comparison of hormone contents in dry fruits of parsnip cultivar Panorama #3 an celery cultivar Victoria. Note that further celery homone results are presented in Suppl. Fig. S4. For gibberellins (GA), only the bioactive metabolites are presented, and a full comparison of all GA metabolites is presented in Suppl. Fig. S5. **e** The effects ABA, GA (GA_4+7_) and fluridone (FLU, carotenoid and thereby ABA biosynthesis inhibitor) on the germination of parsnip Panorama #3 fruits imbibed at the conditions indicated. Mean ± SE values are presented from triplicate plates each with 30 parsnip fruits

To investigate if the observed mechanical pericarp constraint to germination (Fig. 3) was combined with chemical constraint by germination inhibitors we compartively analysed parsnip fruits and (true) seeds for their hormone contents (Fig. 4a, b). The germination of imbibed parsnip fruits and seeds was characterised by a rapid early decline of the high abscisic acid (ABA), indole-3-acetic acid (IAA), and oxylipins jasmonate (JA), JA-Ile, and especially *cis*-OPDA contents (Fig. 3b, c). The general patterns of this decline were very similar to what was observed for celery fruits (Supplementary Fig. S4 and Walker et al. 2021). Roughly similar contents of ABA and IAA were found in parsnip and celery embryo and endosperm tissues (Fig. 3 and Supplementary Fig. S4). In contrast to this, the ABA and IAA contents in parsnip pericarp were > fourfold and ~ tenfold higher compared to the celery pericarp. Very high contents of the oxylipins jasmonate (JA) and JA-Ile, and especially *cis*-(+)−12-oxo-phytodienoic acid (*cis*-OPDA), were detected in dry parsnip fruits and seeds and declined during early imbibition (Fig. 3b–d). In contrast to parsnip, oxylipin contents were very low in dry celery fruits (Fig. 3d, Supplementary Fig. S4).

A comparison GA metabolites between whole fruits of parsnip and celery demonstrated that bioactive GA_4_ and GA_6_ contents were higher in parsnip, the combined bioactive gibberellins GA_1_, GA_3_, GA_4_, and GA_7_ contents are ~ sixfold higher in parsnip fruits as compared to celery, and ~ ninefold if also GA_5_ and GA_6_ were considered (Fig. 3d, Supplementary Fig. S5). The GA:ABA ratio was 1:28 for parsnip fruits, while it was 1:520 for celery fruits. In contrast to celery fruits (Walker et al. 2021), parsnip fruits where not sensitive to GA treatment (Fig. 3e) and they therefore differed in their hormonal regulation. In agreement with a role of pericarp-derived ABA, the germination of parsnip fruits was very sensitive to ABA treatment, and neither affected by fluridone (FLU), a carotenoid and therefore also ABA biosynthesis inhibitor, nor GA (Fig. 4e). While the parsnip pericarp *cis*-OPDA contents were ~ 25 µmol ⋅ g^−1^ dry weight (DW), the *cis*-OPDA contents of celery were ~ 400,000,000-fold lower at ~ 0.06 pmol⋅g^−1^ DW (Fig. 3c). ABA and *cis*-OPDA are known seed germination inhibitors (Linkies and Leubner-Metzger 2012; Dave et al. 2016; Nakabayashi et al. 2022) and the pericarp could therefore confer chemical dormancy to parsnip fruits, while in addition it may act as mechanical constraint in all Apiaceae species.

### Germination, embryo size and pericarp constraint in thermal responses of cultivated and wild Apiaceae species

**Fig. 5:**
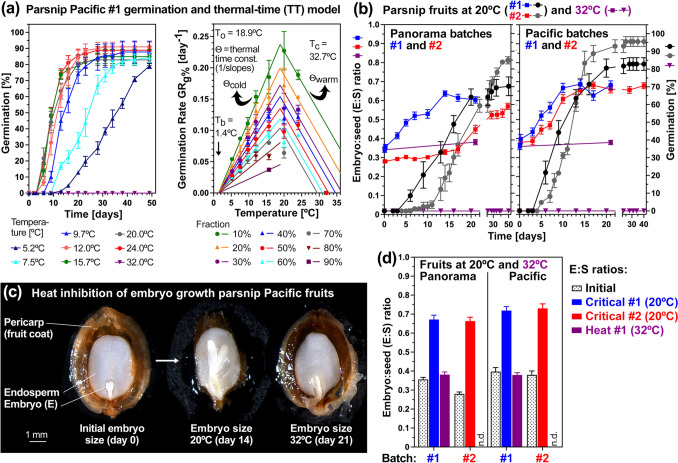
Thermal responses and comparative analysis of critical embryo size and the effect of heat on embryo growth in parsnip cultivars. **a** Responses to different imbibition temperatures (*left panel*) and population-based thermal-time threshold modelling (*right panel*) of parsnip cultivar Pacific batch #1 germination in continuous white light. The thermal-time model delivered estimated cardinal temperatures *T*_b_ (base), *T*_o_ (optimal), *T*_c_ (ceiling or maximal) in °C and the thermal-time constants Θ_cold_ and Θ_warm_ in °C⋅days (derived fom the slopes of the regression lines; see the Material and method section for details) which are indicated in the figure and listed in Table 1. **b** Germination and embryo growth inside fruits imbibed at 20 °C or 32 °C in continuous white light of two independent batches (#1 and #2) of the parsnip cultivars Panorama and Pacific. **c** The effect of heat on the embryo growth inside imbibed fruits of the parsnip cultivar Pacific. **d** Initial and critical embryo:seed (E:S) size ratios inside imbibed fruits of two independent batches (#1 and #2) of parsnip cultivars and the effect of heat on embryo growth. Mean ± SE E:S values are presented for 50 embryos (embryo and seed (excluding pericarp) size were measured); mean ± SE germination values presented are from triplicate plates, each with 30 parsnip fruits

**Fig. 6:**
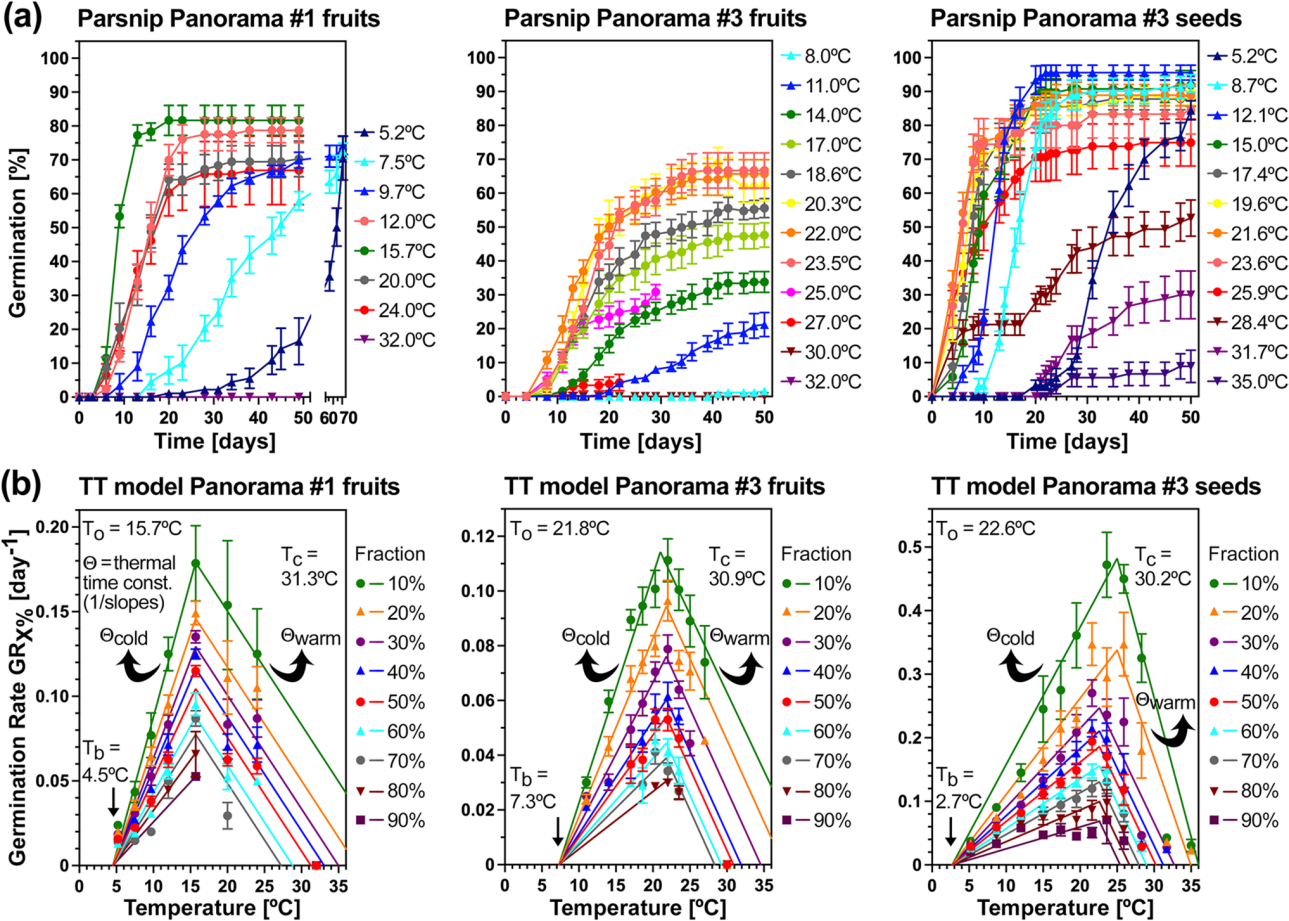
Fruit and seed temperature responses of parsnip cultivar Panorama germination. **a** The effect of different imbibition temperatures on the germination of Panorama fruits (batches #1 and #3) and (true) seeds (batch #3) in continuous white light. The completion of germination by radicle emergence was scored over time and differed considerably also in the maximal germination percentages (*G*_max_) obtained when the fruit batches (#1 versus #3) or for batch #3 fruits and seeds were compared. Mean ± SE values are presented of triplicate plates each with 30 fruits or seeds. **b** Population-based thermal-time (TT) threshold modelling of corresponding fruit and seed germination. The TT models delivered estimated cardinal temperatures *T*_b_ (base), *T*_o_ (optimal), *T*_c_ (ceiling or maximal) in °C and the thermal-time constants Θ_cold_ and Θ_warm_ in °C⋅days (derived from the slopes of the regression lines; see the material and method section for details), which are indicated in the figure and listed in Table 1. Note that pericarp removal to obtain true seeds resulted in increased *G*_max_, enhanced germination speed and reduced *T*_b_, but did not appreciably affect *T*_o_ and *T*_c_

**Fig. 7:**
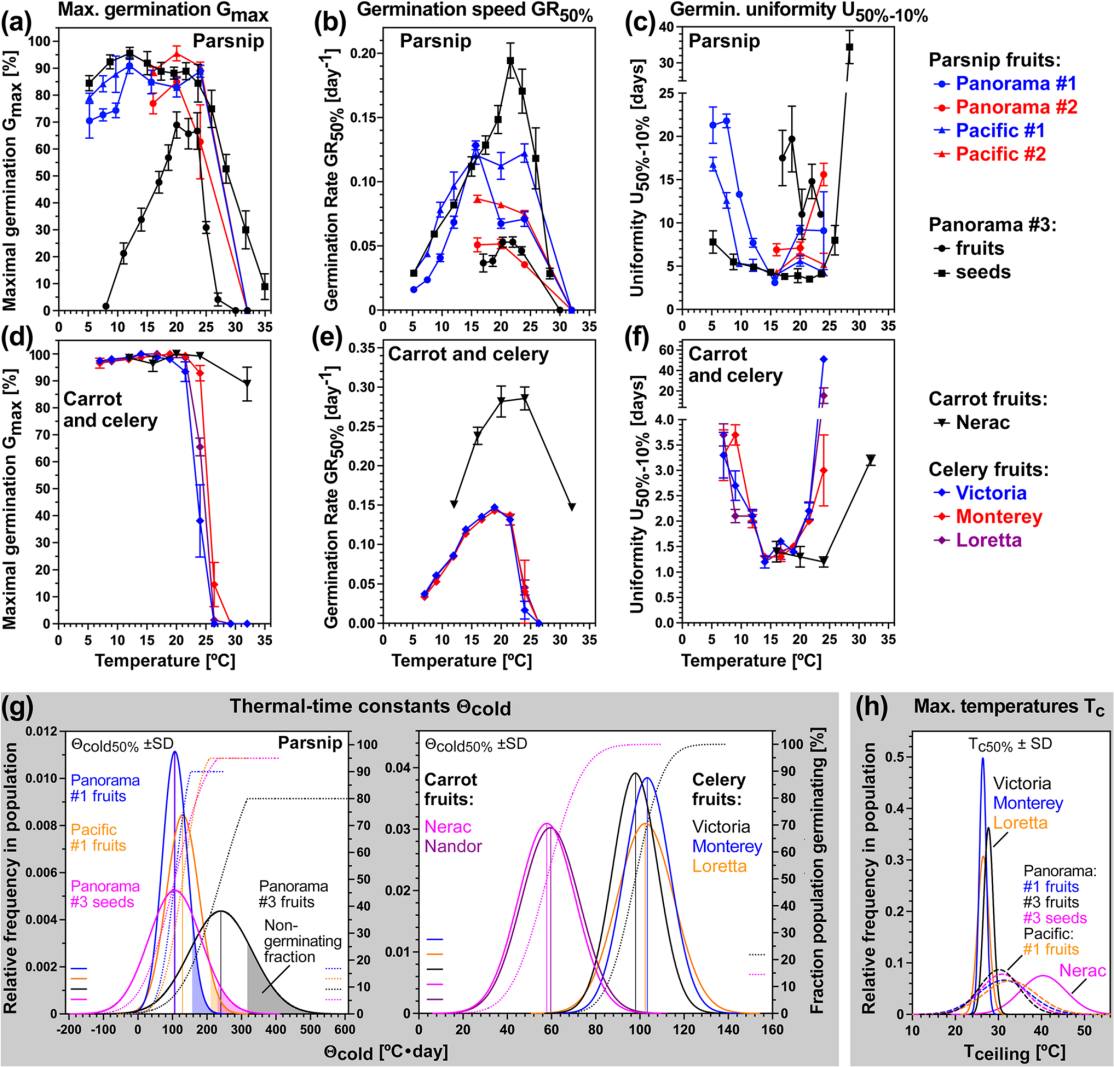
Cross-species comparison of Apiaceae cultivar fruit and seed thermal germination responses. **a** The effect of imbibition temperature on the maximal germination percentages (*G*_max_) of parsnip cultivars (Panorama, Pacific) and batches (#1, #2, #3) fruits and (for batch #3) seeds. **b** The effect of imbibition temperature on parsnip germination speed expressed as germination rates GR_50%_ (inverse of the time required for the populations to reach 50% germination). **c** The effect of imbibition temperature on parsnip germination uniformity *U*_50–10%_ which is the time difference for the populations to reach 10% and 50% germination; note that low values therefore indicate high germination uniformity. **d** The effect of imbibition temperature on *G*_max_ of celery and carrot cultivars. **e** The effect of imbibition temperature on GR_50%_ of celery and carrot cultivars. **f** The effect of imbibition temperature on *U*_50–10%_ of celery and carrot cultivars. **g** Cross-species comparison of frequency distribution of the thermal-time constants Θ_cold50%_ ± SD for parsnip (fruits and seeds), carrot (fruits) and celery (fruits cultivars and batches. Note that non-germinating parsnip fractions are indicated and that for the Panorama #3 batch pericarp removal is associated with increased *G*_max_ (removing the pericarp constraint) and a shift of Θ_cold50%_ ± SD to lower thermal-times. **h** Cross-species comparison of frequency distribution of maximal temperatures *T*_c50%_ ± SD for germination. The obtained cardinal temperature and thermal-time constant values are compiled in Table 1

Earlier work with celery fruits demonstrated that distinct hormone-temperature interactions and molecular mechanisms in response to non-optimal cold and warm temperatures affect pre-germination embryo growth (Walker et al. 2025). We followed this up by comparatively investigating these responses and mechanisms across a gradient of temperatures in different parsnip, carrot and celery cultivars, and compared these to wild Apiaceae species (Figs. 5, 6 and 7, Table 1). Fruits of the parsnip cultivars Panorama #1 (Fig. 5a, *left panel*) and Pacific #1 (Fig. 6a, *left panel*) batches germinated fully (*G*_max_ ~ 85%) between 5.2 and 24 °C, and did not germinate at 32 °C. In contrast to this, the fruits of the Panorama #3 batch only germinated between 11 and 27 °C, and reached a ~ 70% *G*_max_ only in their optimal temperature range between 20 and 24 °C (Fig. 6a, *middle panel*). Pericarp removal enabled the resultant Panorama #3 seeds to germinate fully (*G*_max_ ~ 90%) across a wide temperature window between 5.2 and 24 °C, and to a considerable percentage also in the supra-optimal temperature range between 26 °C (*G*_max_ ~ 70%) and 32 °C (*G*_max_ ~ 30%) (Fig. 6a, *right panel*). To estimate the cardinal temperatures and thermal time constants Θ_cold_ and Θ_warm_, we conducted comparative population-based thermal-time threshold modelling of parsnip fruits and seeds (Figs. 5, 6, Table 1). Parsnip fruits differed in their base temperatures (*T*_b_) ranging between 1.4 °C (Pacific #1) and 7.3 °C (Panorama #3) and optimal temperatures (*T*_o_) ranging between 15.7 °C (Panorama #1) and 21.8 °C (Panorama #3). Panorama #3 fruits differed in the sub-optimal (colder) temperature range from Panorama #1 and Pacific #1 fruits in *G*_max_ and in their germination speed (Germination Rate GR_50%_, i.e. the inverse of the time to reach 50% germination), but were very similar for Θ_cold_ (Figs. 5, 6, Table 1).

**Table 1:** Apiaceae cardinal temperatures (*T*_b_, base; *T*_o_, optimal; *T*_c_, ceiling temperature) and thermal-time constants (Θ) derived from thermal-time modelling of fruit (mericarp) and seed (pericarp manually removed) germination and embryo growth within imbibed fruits

Species	Cultivar or	Batch	Germination	*T*_b_	*T*_o_	Θ_cold50%_ ± SD	*T*_c_ ± SD	Θ_warm_	Source
	wild species		units	[°C]	[°C]	[°C⋅days]	[°C]	[°C⋅days]	
Parsnip	Panorama	#1	Fruits	4.5	15.7	107.0 ± 35.8	31.3 ± 6.0	− 145.2	this work
	Panorama	#2	Fruits	n.d	n.d	n.d	33.0 ± 4.4	− 254.6	this work
	Panorama	#3	Fruits	7.3	21.8	240.5 ± 91.3	30.9 ± 5.1	− 219.9	this work
	Panorama	#3	Seeds	2.7	22.6	106.9 ± 76.0	30.2 ± 4.6	− 41.8	this work
	Pacific	#1	Fruits	1.4	18.9	129.8 ± 47.2	32.7 ± 6.1	− 102.0	this work
	Pacific	#2	Fruits	n.d	n.d	n.d	n.d	− 139.3	this work
Celery	Victoria		Fruits	3.0	19.7	97.9 ± 10.2	27.7 ± 1.1	− 45.0	this work
	Victoria^a^		Fruits	3.0	21.3	86.8 ± 10.1	27.0 ± 0.8	− 40.5	Walker et al. (2025)
	Monterey		Fruits	3.3	20.6	103.3 ± 10.4	26.4 ± 0.8	− 35.7	this work
	Loretta		Fruits	3.0	19.3	102.5 ± 12.9	26.5 ± 1.3	− 47.5	this work
Carrot	Nerac		Fruits	3.1	21.8	57.9 ± 12.9	40.4 ± 5.3	− 57.7	this work
	Nominator		Fruits	2.6	21.9	n.d	~ 27.8	n.d	this work
	Nandor^a^		Fruits	2.1	23.4	59.5 ± 13.2	n.d	n.d	Finch-Savage et al. (1998)
	3-cultivar mean		Fruits	~ 1.7	~ 25.0	n.d	~ 43.3	n.d	(Corbineau et al. 1995)
*Cyclospermum leptophyllum*	Wild species		Fruits	2.7	20/10	219 ± 303	~ 37.0	n.d	Walck et al. (2008)^b^
*Glehia littoralis*	Wild species		Fruits	1.7	≤ 20	140.2 ± 51.6	n.d	n.d	Yeom et al. (2021)^b^
*Glehia littoralis*	Wild species		Seeds	1.7	≤ 20	80.1 ± 34.1	n.d	n.d	Yeom et al. (2021)^b^
*Conopodium majus*	Wild species		Fruits	− 4.4^c^	4.0^c^	n.d	15.1^c^	n.d	(Blandino et al. 2025)^c^
*Aegopodium podagraria*	Wild species		Fruits	− 5.9	~ 15	29.1 ± 18.5	~ 42.7	− 39.2	(Phartyal et al. 2009)^b^
*Ferula gummosa*	Wild species		Fruits	− 2.0	12.5	170.2 ± 94.6	~ 24.5	− 41.0	Zardari et al. (2019)^b^

^a^Germination analysis for the thermal-time modelling was not performed on a temperature gradient table, but in growth cabinets

^b^The germination kinetics from these publications were used to estimate the cardinal temperatures and thermal time constants from MD fruits; for MPD fruits this was after the physiological dormancy component was released by after-ripening or cold-stratification

^c^Means of 9 accessions, temperature ranges [ºC]: *T*_b_ − 6.7 to − 2.6, *T*_o_ 2.5 to 5.3, *T*_c_ 12.1 to 20.5

Comparison of Panorama #3 fruits and seeds revealed that pericarp removal affected the temperature responses by reducing *T*_b_ from 7.3 to 2.7 °C (Fig. 6b), by increasing *G*_max_ and GR_50%_ to Panorama #1 fruit levels in the sub-optimal temperature range (Figs. 5a, 6b, 7a), by increasing GR_50%_ ~ twofold at optimal temperatures without changing *T*_o_ (Figs. 5a, 6b, 7b), and by increasing germination uniformity across the entire sub-optimal and optimal temperature range (Fig. 7c). The thermal time constant Θ_cold-50%_ ± SD = 241 ± 91 °C⋅days for Panorama #3 fruits was reduced > twofold by pericarp removal to 107 ± 76 °C⋅days for Panorama #3 seeds (Fig. 7g, Table 1). Pericarp removal shifted the Panorama #3 Θ_cold-50%_ towards values similar to Panorama #1 and Pacific #1 fruits (Table 1). In contrast to these changes in the sub-optimal and optimal temperature range, pericarp removal did not appreciably affect *T*_c-50%_ ± SD, which was ~ 31 °C for all parsnip cultivars (Fig. 7h, Table 1). However, pericarp removal increased *G*_max_ and GR_50%_ in the supra-optimal temperature range (Figs. 6b, 7a). As for parsnip, pericarp removal also enhanced *G*_max_ and germination speed of the wild Apiaceae species *Glehia littoralis* (Yeom et al. 2021) and reduced Θ_cold-50%_ ~ twofold (Table 1). In contrast to parsnip, pericarp removal of *G. littoralis* did not affect *T*_b_ and therefore did not widen the permissive sub-optimal temperature range for germination (Table 1). Taken together, these results suggest that the role and importance in thermal responses of the pericarp as a mechanical constraint and source of germination-inhibiting chemicals differs between Apiaceae species.

Thermoinhibition of parsnip fruit germination at 32 °C was caused by completely blocking pre-germination embryo growth to reach the critical E:S ratio required for the completion of germination (Fig. 5b–d). In contrast to parsnip, fruits of the carrot cultivar Nerac germinated readily at 32 °C (Supplementary Fig. S6). Consistent with this, Nerac’s *T*_c-50%_ (40.4 °C) was much higher compared to parsnip (~ 31 °C) and celery (~ 27 °C) (Fig. 7h, Table 1). Fruits of three celery cultivars germinated fully (*G*_max_ ~ 95%) between 7.0 and 21.5 °C, differed at 24–26.4 °C, and did not germinate above 29.2 °C (Supplementary Fig. S7). Germination rates across different temperatures of carrot were higher for carrot as compared to celery and parsnip (Fig. 7b,e), and germination uniformity of carrot was better across a broader temperature range as compared to celery (Fig. 7f). Thermal-time modelling demonstrated that the cardinal temperatures of celery fruit germination of the Victoria, Monterey and Loretta cultivars were very similar (Supplementary Fig. S7). For Victoria fruits these were *T*_b_ = 3.0 °C, *T*_o_ = 19.4 °C, *T*_c_ = 26.6 °C, and Θ_cold-50%_ ± SD = 99 ± 10 °C⋅days (Fig. 7g, Table 1). The thermal-time constants Θ_cold-50%_ of the three celery cultivars were very similar to parsnip Panorama #1 fruits and Panorama #3 seeds (Fig. 7g, Table 1). Compared to celery and parsnip, Θ_cold-50%_ of the carrot cultivars Nerac and Nandor were much lower (Fig. 7g, Table 1).

Our comparative analysis of the thermal responses of three Apiaceae crops and several cultivars therefore demonstrates that the differences in cardinal temperatures are mainly species-specific and that within a crop species cultivar and production environment (batch) provide further variation (Fig. 7, Table 1). For the sub-optimal temperature range, the thermal-time constants Θ_cold-50%_ and Θ_warm_, as well as *T*_c_ differed mainly in a species-specific manner, whereas *T*_b_ was very similar between the three Apiaceae species. For cultivated carrot, very similar cardinal temperatures and thermal-time constants compared to the Nerac cultivar (Table 1) were also obtained by other researchers (Corbineau et al. 1995; Finch-Savage et al. 1998; Nascimento et al. 2013). In contrast to the Apiaceae crops, which mainly have MD, most wild Apiaceae species have MPD. When the physiological component of MPD fruits of wild Apiaceae species was experimentally released by after-ripening or stratification, they resemble MD fruits for which we conducted population-based thermal-time threshold modelling (Table 1) using the published germination kinetics for *Cyclospermum leptophyllum* (Walck et al. 2008), *Glehia littoralis* (Yeom et al. 2021), *Aegopodium podagraria* (Phartyal et al. 2009) and *Ferula gummosa* (Zardari et al. 2019). Table 1 also presents the mean values of nine *Conopodium majus* (Blandino et al. 2025). The two general conclusions from this comparison of the thermal responses between Apiaceae crops and wild species are that (i) MPD fruits of wild Apiaceae species therefore require in addition a cold or warm stratification period to break the physiological dormancy component of the MPD, and (ii) compared to MD fruits of cultivated Apiaceae species, the germination window of wild Apiaceae species is shifted towards colder temperatures (see discussion).

## Discussion

### Pericarp constraint mechanisms to Apiaceae germination and hormonal control of germination

**Fig. 8:**
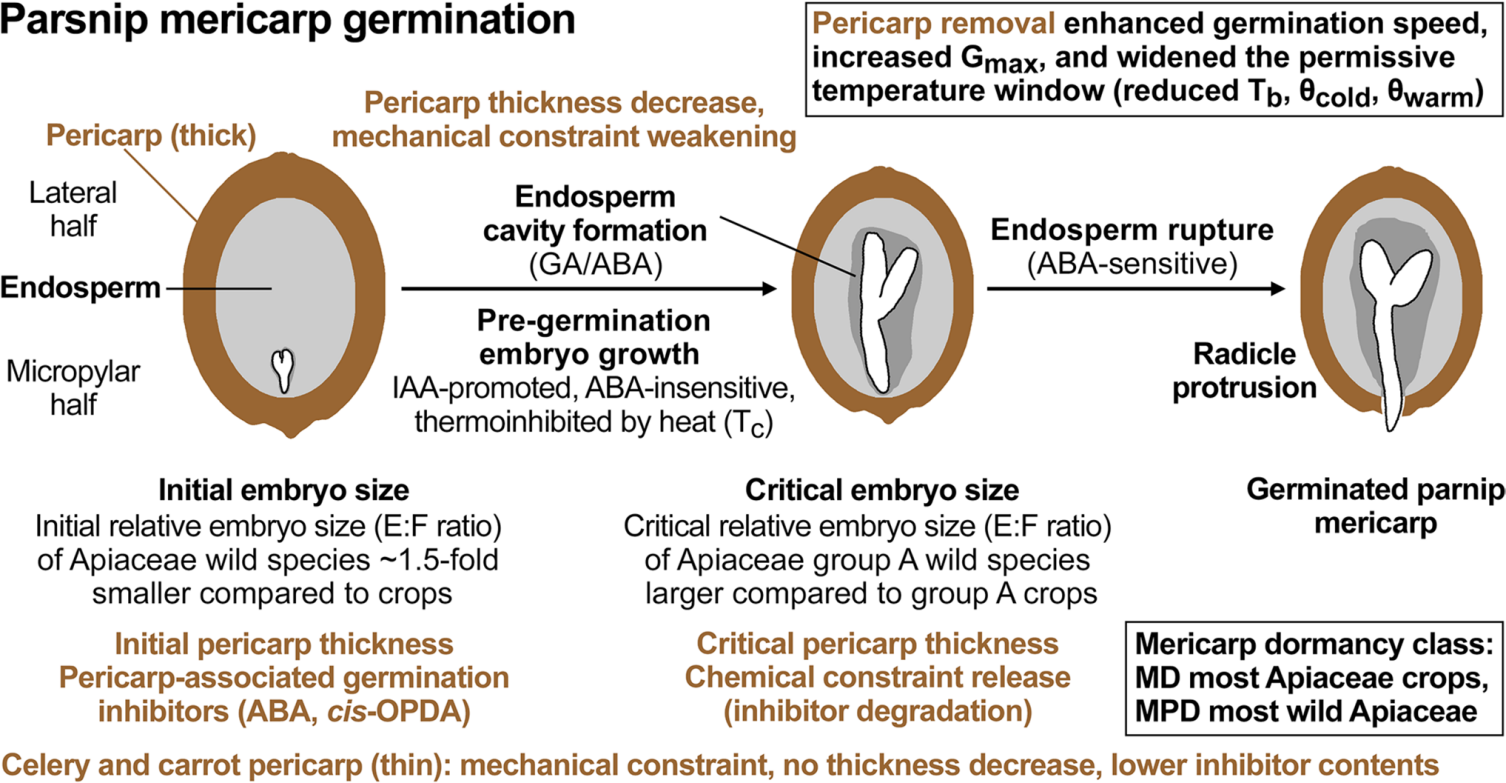
Schematic summary of key findings for Apiaceae mericarp germination with the focus on parsnip. Note that parsnip mericarps are characterised by a thick pericarp (~ 750 µm) which decreases in thickness during imbibition. In contrast to this, celery, carrot and many other Apiaceae crops have thin pericaps (~ 50 to ~ 150 µm) which to not appreciably change in their thickness during imbibition. Note further that parsnip and celery pericarps differ in their hormonal germination inhibitor contents; for further details see main text

We found by pericarp ablation, morphological, biomechanical and hormonal analysis that, in addition to its role in distinct dispersal strategies (Liu et al. 2006, 2012; Wojewodzka et al. 2019), the pericarp of Apiaceae mericarps (hereafter fruits) acts as an important germination constraint and restricts the permissive temperature window for embryo growth and the completion of germination by radicle emergence (see schematic summary, Fig. 8). Apiaceae pericarp thickness varies considerably between species: For the crop cultivars of fennel, dill and cumin ~ 75 µm, ~ 45 µm and ~ 65 µm were reported (Ma et al. 2015; Soltani et al. 2019). Parsnip has a thick pericarp and its thickness decreased during imbibition in parallel with pre-germination embryo growth (Fig. 8). This decrease in pericarp thickness was ~ 1.3-fold at the time when the critical embryo size was reached, which is required for the completion of germination by radicle emergence. Biomechanical analysis demonstrated that, associated with this, the pericarp tissue strength (puncture force) of imbibed parsnip fruits decreased significantly in association with the decrease in pericarp thickness. In contrast to parsnip (~ 750 µm), the thinner pericarps of celery (~ 50 µm) and carrot (~ 150 µm) did not appreciably decrease in thickness during fruit imbibition (Fig. 3). The parsely pericarp is ~ 200 µm and during extended priming the softer exocarp and mesocarp tissues were increasingly degraded (Olszewski et al. 2004, 2005).

The endocarp of many Apiaceae species contains condensed phenols and/or lignin (Olszewski et al. 2005; Liu et al. 2006; Atia et al. 2010; Ma et al. 2015; Mustafina et al. 2021). Apiaceae species differed in pericarp properties such as the number of lignified endocarp layers, with parsnip belonging to the Apiaceae species that contain several lignin-containing endocarp layers (Fig. 3). The mean ± SE pericarp thickness of 14 wild carrot species was 53.4 ± 8.4 µm (range 28–132 µm), while four other wild species of group B had pericarp thicknesses between 81 and 150 µm (Kadluczka and Grzebelus 2022). Pericarp removal (ablation) resulted in > twofold faster embryo growth inside the endosperm to reach the critical size for radicle emergence to complete germination (Fig. 3). Ablation experiments of the celery demonstrated that the micropylar pericarp constitutes a germination constraint as its removal increased germination speed and uniformity (Fig. S3). The constraint to germination by the pericarp therefore seems to have a mechanical component, but it does not block or delay seed water uptake as demonstrated for the pericarp of several Brassicaceae species (Cousens et al. 2010; Mamut et al. 2014; Zhou et al. 2015; Sperber et al. 2017; Steinbrecher et al. 2025). The pericarp may also act as a barrier to oxygen uptake into the seed as it was demonstrated for *Beta vulgaris* (Coumans et al. 1976; Richard et al. 1989; Hermann et al. 2007), *Aethionema arabicum* (Chandler et al. 2024), and work with carrot by Corbineau et al. (1995) suggests this may also maybe the case for Apiaceae.

The mericarps of the halophyte *Crithmum maritimum* (Apiaceae group A) were adapted to long-term floating capacity due to a thick spongy mesocarp (~ 300 µm), while the endocarp was very thin (Atia et al. 2010). Pericarp removal increased *G*_max_ and germination speed ~ twofold (Atia et al. 2010), but the mechanisms of the pericarp constraint were not investigated. Pericarp removal also increased *G*_max_ of parsnip cultivar Panorama batch #3 from ~ 70% to ~ 95% (Fig. 5), which is in contrast to *Petroselinum crispum* (parsley, Apiaceae) fruits where scarification did not increase *G*_max_ (Hassell and Kretchman 1997). These authors also demonstrated that leachates from Apiaceae fruits inhibited the germination of Apiaceae fruits and radish seeds with parsnip leachates being most effective. Thermoinhibition caused by imbibing celery fruits in the dark at high temperatures is known to be associated with the accumulation of germination inhibitors for which ABA, phthalides and terpenoids were proposed (Thomas et al. 1986; Ma et al. 2015; Önder et al. 2024). Germination inhibition by pericarp wash-water containing ABA, salts (NaCl) and phenolic compounds is also known for sugarbeet (Hermann et al. 2007; Ignatz et al. 2019) and invasive *Lepidium draba* (Mohammed et al. 2019).

We demonstrate here that very high contents of hormonal inhibitors such as ABA and *cis*-OPDA are present in parsnip pericarp tissue (Figs. 4, 8). ABA contents in parsnip pericarp were > threefold higher compared to celery pericarp (this work and Walker et al. 2021), but >  > 200-fold lower compared to sugar beet, sunflower and *Lepidium draba* pericarp (Ignatz et al. 2019; Mohammed et al. 2019; Del Bel et al. 2023). *cis*-OPDA is known for its control of *Arabidopsis thaliana* seed dormancy (Dave et al. 2016) and in sunflower dormancy maintenance it counteracts the dormancy-breaking effects of cytokinins (Del Bel et al. 2023). Sunflower pericarp contains ~ 20,000 pmol⋅g^−1^ DW *cis*-OPDA, which is very similar to the ~ 25,000 pmol⋅g^−1^ DW *cis*-OPDA we quantified in parsnip pericarp. In contrast to parsnip, *cis*-OPDA contents of celery pericarp are very low (Figs. 4, S4). Pericarp removal enhanced parsnip germination speed, while 50% germination of parsnip fruits required ~ 3 weeks, it took only ~ 1 week for parsnip seeds and celery fruits. It is, therefore, possible that in addition to mechanical restraint weakening, leaching of *cis*-OPDA and ABA play roles in the pericarp-mediated delay of parsnip fruits.

Walker et al. (2021) identified a complex interaction between GA, IAA, and ABA metabolism and changes in the tissue-specific sensitivities to these hormones controls the unique germination programme of MD celery fruits. A similarly complex interaction is supported and a unique germination programme is also evident for MD parsnip fruits (Fig. 4) and confirms, for example the importance of the IAA/ABA ratio in MD fruits. As for celery, also for parsnip the IAA and ABA contents declined during imbibition, but in both species the IAA/ABA ratios increased. The IAA/ABA ratios also increased during carrot fruit germination (Tan et al. 2923). ABA does not inhibit pre-germination embryo of MD crops and auxins (IAA) play cardinal roles in the promotion of embryo cell expansion and division associated with the pre-germination embryo (this work and Van der Toorn and Karssen 1992; Homrichhausen et al. 2003; Walker et al. 2021, 2025). Our work revealed important roles for IAA not traditionally associated with physiologically dormant (PD) seeds. In *A. thaliana* seeds auxin controls seed dormancy through stimulation of ABA signalling (Liu et al. 2013; Yan et al. 2014; Chahtane et al. 2017) and is known as a key regulator of dormancy, longevity and maturation in PD seeds (Shu et al. 2016; Matilla 2020; Pellizzaro et al. 2020), and auxin homeostasis is also important for the germination of non-dormant seeds (Nakabayashi et al. 2022). Auxin transport appears to be important for Apiaceae seed development (Koryzniene et al. 2019) and germination 20der optimal conditions, it promoted dill fruit germination upon salt stress (Unver and Tilki 2012) and enhanced size, germination percentages and vigour of carrot fruits (Noor et al. 2020). As for celery and parsnip, the IAA contents also increased during the late germination phase of carrot, and this was accompanied by the increased expression of IAA biosynthesis genes (this work and Walker et al. 2021, 2025; Tan et al. 2023).

Beyond ABA and IAA, also jasmonates and their *cis*-OPDA precursor play important roles in seed dormancy and germination of PD seeds (Linkies and Leubner-Metzger 2012; Dave et al. 2016; Pan et al. 2023). In general, jasmonates promote dormancy and inhibit germination through crosstalk with other phytohormones. We showed here that jasmonic acid (JA) and the bioactive JA-Ile contents declined in parsnip and celery fruits during imbibition (Figs. 4, S4). While the JA/ABA ratios increased, as the IAA/ABA ratios, the JA-Ile/ABA ratios decreased. Further to this, the tissue-specific JA and JA-Ile contents differed between parsnip and celery. The importance of these findings and the roles of jasmonates in MD/MPD seeds are not known and require future research. Taken together, the pre-germination embryo growth inside the fruits of Apiaceae species is not simply the completion of embryogenesis or *A. thaliana* equivalent post-embryogenesis growth, but a distinct process as revealed by the hormonal mechanisms, embryo-endosperm interactions, pericarp mechanical and chemical constraint and the spatiotemporal expression patterns of corresponding genes (this work and Van der Toorn and Karssen 1992; Homrichhausen et al. 2003; Walker et al. 2021, 2025).

### Pericarp constraint and embryo growth control Apiaceae dormancy and thermal responses

The physiological component of the morphological dormancy classes is usually lost in horticultural Apiaceae crops, including carrot (Homrichhausen et al. 2003; Nascimento et al. 2013) and celery (Biddington and Thomas 1978; Walker et al. 2021), and these species consequently have MD. Ecophysiology studies suggest that there is a very close association between MD and MPD with numerous species capable of exhibiting both dormancy types, as well as multiple levels of MPD, within a single species, population or plant. For example, *Pastinaca sativa* (wild parsnip) produce mixtures of MPD and MD mericarps (Baskin and Baskin 1979). Only ~ 25% germination was observed in the freshly harvested state, and this increased during 3 months after-ripening to ~ 75% of the mericarps germinating. We found that our parsnip cultivar Panorama #3 batch was a mixture between ~ 70% of MD (*G*_max_ of fruit germination) and ~ 25% MPD mericarps and that pericarp removal resulted in ~ 95% germination (*G*_max_ of seed germination). The pericarp removal was associated with increased germination speed and with widening of the permissive temperature window (Figs. 5, 6, 8). As a result of the parsnip pericarp removal, *T*_b_ was lowered by > 4ºC, and this was without appreciably affecting *T*_o_ and *T*_c_. In contrast to parsnip, pericarp removal in *Glehia littoralis* (Yeom et al. 2021), did not affect *T*_b_ (Table 1). While the germination speed of *G. littoralis* seeds was increased compared to fruits, the thermal profile remained unchanged. This lowered *T*_b_ of parsnip seeds (pericarp removed) was very similar to the *T*_b_ of celery and carrot fruits (Table 1), suggesting that the pericarp constraint plays a larger role in parsnip as compared to celery and carrot. This difference could be simply caused by removing the *cis*-OPDA inhibitor together with the parsnip pericarp, but it also demonstrates that embryo growth inside parsnip seeds needs to reach a critical size required for germination must also occur upon chilling.

It seems that the mechanisms by which cold temperatures delay Apiaceae germination are by reducing, but not blocking, the embryo growth rate, and that this constitues a conserved mechanism (Walker et al. 2025). In contrast to the sub-optimal (colder) temperature range, supra-optimal (warmer) temperatures caused thermoinhibition of parsnip (Fig. 5) and celery (Walker et al. 2025) fruits by blocking embryo growth at 32 °C and 29 °C, respectively. The embryo growth inhibition by supra-optimal temperatures is, however, not simply an ABA effect because treatment of celery fruits imbibed at optimal temperature with 100 µM ABA did not appreciably inhibit embryo growth inside the fruit to reach the critical size required for radicle protrusion (Walker et al. 2021). Abscisic acid (100 µM) also did not inhibit embryo growth inside carrot fruits imbibed at 25 °C (Homrichhausen et al. 2003), and carrot fruit germination was not blocked at 32 °C (this work, and Corbineau et al. 1995; Nascimento et al. 2013). Embryo growth inside imbibed parsnip fruits was blocked at 32 °C resulting in blocked fruit germination (Fig. 5). Pericarp removal of the parsnip Panorama #3 batch did not appreciably change *T*_c_, but resulted in increased germination percentage of imbibed parsnip seeds at 32 °C (~ 30%). Taken together this suggests that removal of the pericarp constraint (including the stored ABA and *cis*-OPDA inhibitors) may remove this block and permit reduced rates of endosperm degradation and embryo growth also upon heat stress.

### Thermal responses and relative embryo sizes differ between wild and cultivated Apiaceae species

In contrast to Apiaceae crops which mainly produce MD fruits, most wild Apiaceae species (Fig. 2, Supplementary Table S2) produce MPD fruits (Fig. 8). MPD first require a warm stratification (≥ 15 °C) and/or cold stratification (0–10 °C) period to break the physiological dormancy component (Baskin and Baskin 201, 2021; Zhang et al. 2019a; Visscher et al. 2022; Carta et al. 2024). This stratification period is usually several weeks and may coincide with embryo growth. In cases of simple MPD either warm (≥ 15 °C) or cold (0–10 °C) stratification is required as dormancy-breaking pretreatment, and subsequent embryo growth requires warm temperatures (≥ 15 °C). Examples for wild Apiaceae species with non-deep simple MPD that require cold stratification as dormancy-breaking pretreatment include *Angelica keiskei*, *Angelica sylvestris, Selinum carvifolia*, and *Cyclospermum leptophyllum* (Vandelook et al. 2007a; Walck et al. 2008; Zhang et al. 2019b). An example for non-deep simple MPD that requires warm stratification as dormancy-breaking pretreatment is *Chaerophyllum tainturieri* (Baskin and Baskin 1990a).

The majority of wild Apiaceae species have complex MPD (Supplementary Table S2 and Visscher et al. 2022). In cases of complex MPD cold (0–10 °C) stratification is required as dormancy-breaking pretreatment, and subsequent embryo growth (from initial 0.04–0.37 to final ~ 0.9 E:F ratio) requires cold temperatures (0–10 °C). In the case of non-deep and intermediate complex MPD, the cold stratification can be replaced by GA treatment. Examples for Apiaceae species with non-deep or intermediate complex MPD (cold stratification temperatures indicated) include *Cicuta virosa* (4 °C), *Elwendia caroides* (5 °C), *Elwendia wolfii* (5 °C), *Ferula gummosa* (< 10 °C), *Conopodium majus* (2.5–5 °C), and *Bunium persicum* (5 °C) (Cho et al. 2018; Zardari et al. 2019; Mohammadianfar et al. 2024; Rahimi et al. 2024; Blandino et al. 2025). In the case of deep complex MPD, the cold stratification cannot be replaced by GA treatment. Several weeks of cold stratification are also required for Apiaceae species with deep complex MPD at the temperatures indicated for physiological dormancy release and embryo growth for *Aegopodium podagraria* (0–5 °C), four *Osmorhiza* species (1–5 °C, no embryo growth at 24/15 °C), *Lomatium dissectum* (3–6 °C), *Ferula ovina* (3–5 °C), *Glehnia littoralis* (5 °C), *Anthriscus sylvestris* (1 °C, no embryo growth at 25/15 °C), and *Chaerophyllum temulum* (5 °C) (Baskin et al. 2000; Walck and Hidayati 2004; Vandelook et al.2007b, 2009; Phartyal et al. 2009; Scholten et al. 2009; Fasih and Afshari 2018; Yeom et al. 2021).

When the physiological component of MPD fruits of wild Apiaceae species was experimentally released by after-ripening or stratification, they resemble MD fruits for which we conducted population-based thermal-time threshold modelling (Table 1) using published germination kinetics. For *Cyclospermum leptophyllum* with the physiological component of the non-deep simple MPD released (Walck et al. 2008), cardinal temperatures and a thermal time constant very similar to parsnip were obtained (Table 1). The other wild Apiaceae species with the physiological component of their complex MPD released (Phartyal et al. 2009; Zardari et al. 2019; Yeom et al. 2021; Blandino et al. 2025), differed from the Apiaceae crops in that their cardinal temperatures were shifted to colder temperatures (Table 1). *T*_b_ was − 5.9 to 1.7 °C, with *Conopodium majus* (− 4.4 °C), nine *Aegopodium podagraria* populations (− 6.7 to − 2.6 °C) and *Ferula gummosa* (− 2.0 °C) having *T*_b_ values below 0 °C. Also their *T*_o_ and *T*_c_ values were considerably lower, *T*_o_ was, for example, 2.5 to 5.3 °C, and *T*_c_ 12.1 to 20.5 °C for the), nine *A. podagraria* populations (Blandino et al. 2025). Germination after release of the physiological component of complex MPD was optimal at 0 °C to 5 °C and inhibited at higher temperatures for *A. podagraria* and the *Osmorhiza* species, optimal at 4 °C and inhibited at 12 °C for *Lomatium dissectum*, optimal at 3–5 °C and inhibited at 10 °C for *Ferula ovina* (Baskin and Baskin 1991; Baskin et al. 1995; Walck et al. 2002; Walck and Hidayati 2004; Scholten et al. 2009; Fasih and Afshari 2018; Blandino et al. 2025). Compared to cultivated celery (*Apium graveolens*, MD), wild *Apium repens* has a similar permissive temperature range for germination, but has MPD (Burmeier and Jensen 2008).

Relative embryo size is a key seed trait for which major shifts occurred early in the evolutionary history of the angiosperms (Carta et al. 2024; Vandelook and Carta 2025). Apiaceae mericarps represent the ancient morphological seed trait characterised by an underdeveloped (small) embryo embedded in abundant living endosperm tissue. For the Apiaceae, it was proposed by Vandelook et al. (2012) that relative initial embryo size evolved as an adaptation to habitat and life cycle, whereas dormancy was mainly related to local temperature. Larger initial relative embryo lengths at dispersal of Apiaceae fruits (mericarps) resulted in increased germination speed in wild carrots as an adaptation to local climate conditions (Vandelook et al. 2024). We showed here that Apiaceae crops (celery, parsnip, dill, fennel, carrot), which were derived from breeding programmes have larger initial relative embryo lengths as compared to their wild relatives. They also have an MD and therefore lost the physiological dormancy component of the MPD fruits dispersed by their wild relatives. On the other hand, Apiaceae landraces such as for cumin still have MPD (Soltani et al. 2019), and wild parsnip plants produce mixtures of MPD and MD mericarps (Baskin and Baskin 1979; Hendrix et al. 1991). As wild parsnip, also *Conium maculatum* produces a mixture of MD and MPD fruits (Baskin and Baskin 1990b). In *Ferula gummosa* 80% of fruits were deep complex MPD and 20% were intermediate complex MPD (Zardari et al. 2019), in *Smyrnium cordifolium,* most fruits were deep complex MPD and a smaller fraction non-deep complex MPD (Zarei-Gavkosh et al. 2022), in *Hladnikia pastinacifolia* fruits with simple and complex MPD were produced at different umbels (Sajna et al. 2019), and in *Sanicula* species, mixtures of fruits with non-deep complex and deep complex MPD were produced (Hawkins et al. 2010).

We established here that in addition to the larger initial relative embryo size of crop compared to the wild Apiaceae, for the group A Apiaceae the mean critical relative embryo size of crop cultivars is smaller compared to the wild group A species. This was especially due to the parsnip (Tordylieae) cultivars with a critical E:F ratio ~ 0.56 (Fig. 2, Supplementary Table S2), and was not only evident when compared to all wild group A species, but also when compared to wild Tordylieae species (critical E:F ratio ~ 0.80). We established further, that there are key role and mechanisms of pericarp properties that differ between Apiaceae mericarps. In parsnip, the thick pericarp provides a mechanical and a chemical constraint to germination due to its high concentrations of hormonal inhibitors (ABA and *cis*-OPDA inhibitors). Mechanical constraint weakening during imbibition includes decrease in pericarp thickness which is associated with pre-germination embryo growth to the critical E:F ratio required for the completion of mericarp germination by radicle protusion. This coat (micropylar endopsperm plus pericarp) dormancy component combined with relative embryo length as the morphological dormancy component are, therefore, key traits of adaptive importance for MD/MPD fruits. Our comparative analysis of between Apiaceae species suggests that the distinction between MD and MPD, as well as between the different levels of MPD, is probably rather fluid. In terms of dormancy terminology, it is the result of a variation in hormonal, mechanical and thermal thresholds which control endosperm degradation, pericarp weakening and within-fruit embryo growth, and thereby the environmental sensitivity of mericarp germination.

## Supplementary Information

Below is the link to the electronic supplementary material.

Supplementary file1 (PDF 3930 KB)

## Data Availability

The data generated in this study are available online in the electronic supplementary material and through figshare: 10.17637/rh.30068110.

## Abbreviations

*cis*-OPDA: *cis*-(+)-12-Oxo-phytodienoic acid
E:F: Embryo:fruit length ratio
E:S: Embryo:seed length ratio
GA: Gibberellin
MD: Morphological dormancy
MPD: Morphophysiological dormancy
PF: Puncture force
