# Supplementary material for: Morphological dormancy, embryo growth and pericarp restraint during crop and wild Apiaceae mericarp germination in response to ambient temperature
Source: Planta. 2025 Nov 1;262(6):142. doi: 10.1007/s00425-025-04850-7 (PMC12579680; doi:10.1007/s00425-025-04850-7)
Supplement: Supplementary file 1 — Supplementary file1 (PDF 3930 KB) [file 425_2025_4850_MOESM1_ESM.pdf]

Morphological dormancy, embryo growth and pericarp restraint during crop and wild Apiaceae mericarp germination in response to ambient temperature

Kazumi Nakabayashi[1,2], Lena Fatelnig[1], Matthew Walker[1,3], Sue Kennedy[4], James E. Hourston[1,5], Ondřej Novák[6], Danuše Tarkowská[6], Miroslav Strnad[6], Frances Gawthrop[3], and Tina Steinbrecher[1,*], Gerhard Leubner-Metzger[1,6,*]

[1] Department of Biological Sciences, Royal Holloway University of London, Egham, Surrey, TW20 0EX, United Kingdom, Web: 'The Seed Biology Place' - www.seedbiology.eu

[2] Department of Agro-environmental Science, Obihiro University of Agriculture and Veterinary Medicine, Obihiro, Hokkaido, 080-8555, Japan

[3] Tozer Seeds Ltd, Cobham, Surrey, KT11 3EH, United Kingdom

[4] Elsoms Seeds Ltd, Spalding, Lincolnshire, PE11 1QG, United Kingdom

[5] Eden Research plc, Milton Park, Oxfordshire, OX14 4SA, United Kingdom

[6] Laboratory of Growth Regulators, Institute of Experimental Botany, Czech Academy of Sciences and Faculty of Science, Palacký University Olomouc, CZ-78371 Olomouc, Czech Republic

[*] For correspondence: E-mail Gerhard.Leubner@rhul.ac.uk or Tina.Steinbrecher@rhul.ac.uk

Journal: Planta

Supplementary Information

https://doi.org/10.1007/s00425-025-

© The Author(s) 2025. This is an open access article distributed under the terms of the Creative Commons Attribution License (https://creativecommons.org/licenses/by/4.0/) which permits unrestricted reuse, distribution, and reproduction in any medium, provided the original work is properly cited.

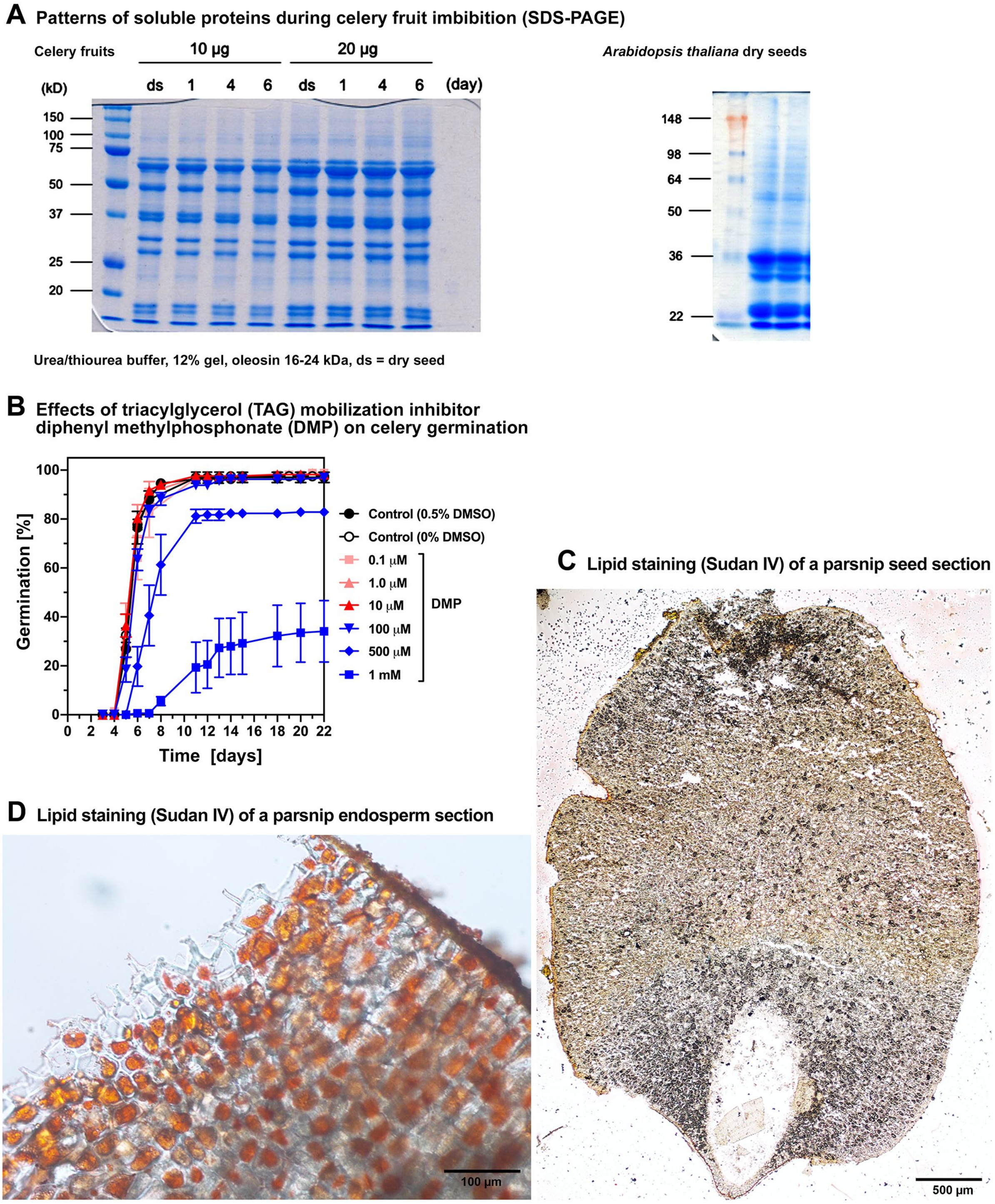

Supplementary Fig. S1. Storage protein and lipid mobilization in celery and parsnip endosperms. **(A)** SDS-PAGE analysis of soluble proteins in the endosperm during *Apium graveolens* (celery) cultivar Victoria fruit imbibition at 20ºC in continuous white light. Note that the celery storage protein pattern differs considerably from *Arabidopsis thaliana* seeds which is shown for comparison.

(B) Effects of the triacylglycerol (TAG) mobilization inhibitor diphenyl methylphosphonate (DMP) on the germination of imbibed Victoria fruits. DMP is known to be a fairly specific inhibitor for TAG mobilization (Brown et al., 2013). Mean ± SEM values are presented of triplicate plates each with 50 celery fruits.

(C-D) Sudan IV histological staining of lipids in *Pastinaca sativa* (parsnip) cultivar Panorama batch #3 endosperm sections. Note thestained oilbodies in the endosperm, while the small embryo in the seed section does not show comparable lipid staining.

Brown, L. A., Larson, T. R., Graham, I. A., Hawes, C., Paudyal, R., Warriner, S. L., & Baker, A. (2013). An inhibitor of oil body mobilization in Arabidopsis. *New Phytologist, 200*, 641-649.

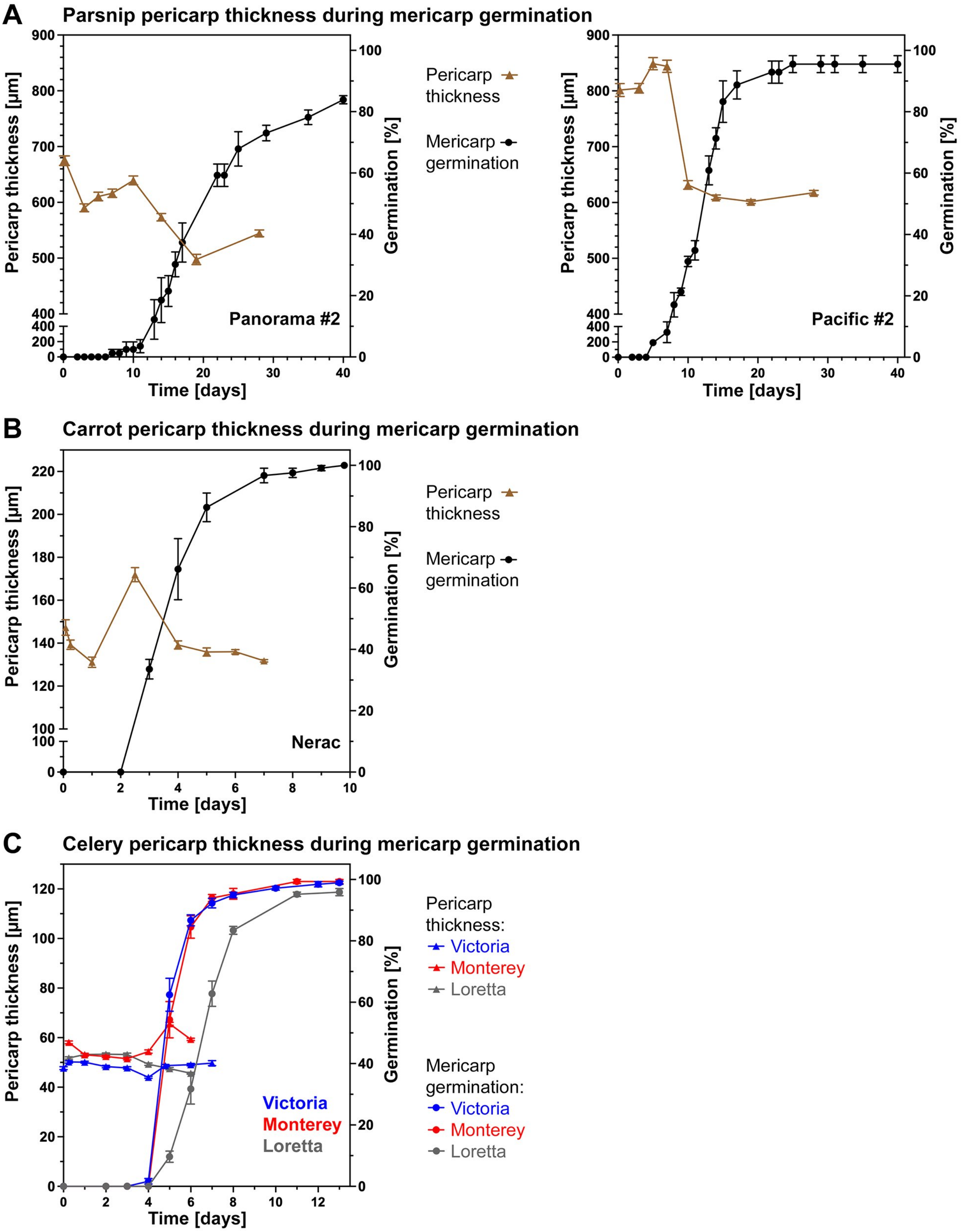

Supplementary Fig. S2. Apiaceae pericarp thickness during mericarp imbibition and germination. **(A)** Pericarp thickness and germination of imbibed parsnip cultivars Panorama and Pacific, seedlots #2. **(B)** Pericarp thickness and germination of imbibed carrot cultivar Nerac. **(C)** Pericarp thickness and germination of imbibed celery cultivars Victoria, Monterey and Loretta. Imbibition conditions: 20ºC, continuous light; Mean ± SEM of three replicate plates each with 30 (parsnip) or 50 (carrot, celery) fruits. Note that the pericarp thickness differs considerably between the crop species.

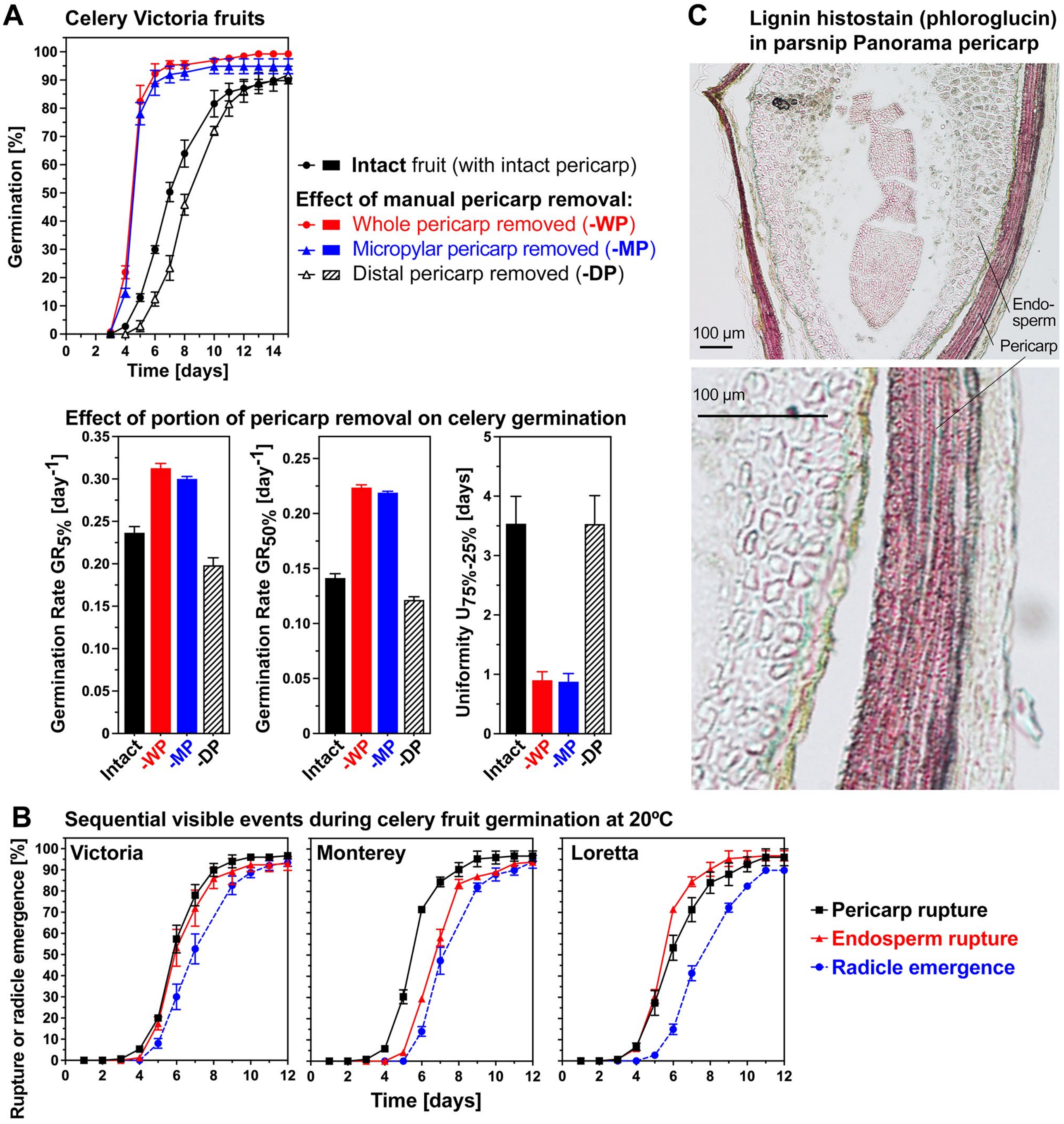

Supplementary Fig. S3. Roles and properties of the Apiaceae pericarp as germination constraint. **(A)** Pericarp ablation experiments with celery cultivar Victoria fruits imbibed (20 ºC in continuous light) with intact (control) pericarp or with a portion (as indicated) of the pericarp removed. Germination, including onset (germination rate $GR_{5\%}$, i.e. the inverse of the time to reach 5% germination) speed ($GR_{50\%}$, i.e. the inverse of the time to reach 50% germination) and uniformity ($U_{75\%-25\%}$ which is the time difference for the populations to reach 25% and 75% germination); note that low $U_{75\%-25\%}$ values therefore indicate high germination uniformity. Note that removal of the micropylar pericarp, but not the distal pericarp, affected celery fruit germination.
(B) Rupture of pericarp and endosperm as sequential visible events during the germination of celery fruits of three cultivars. Mean values ± SEM are presented for triplicate plates each with 50 fruits.
(C) Lignin histostaining (phloroglucinol) of parsnip Panorama #3 fruit sections. Note that the pericarp stains heavily for lignin which is detected in different layers of the pericarp.

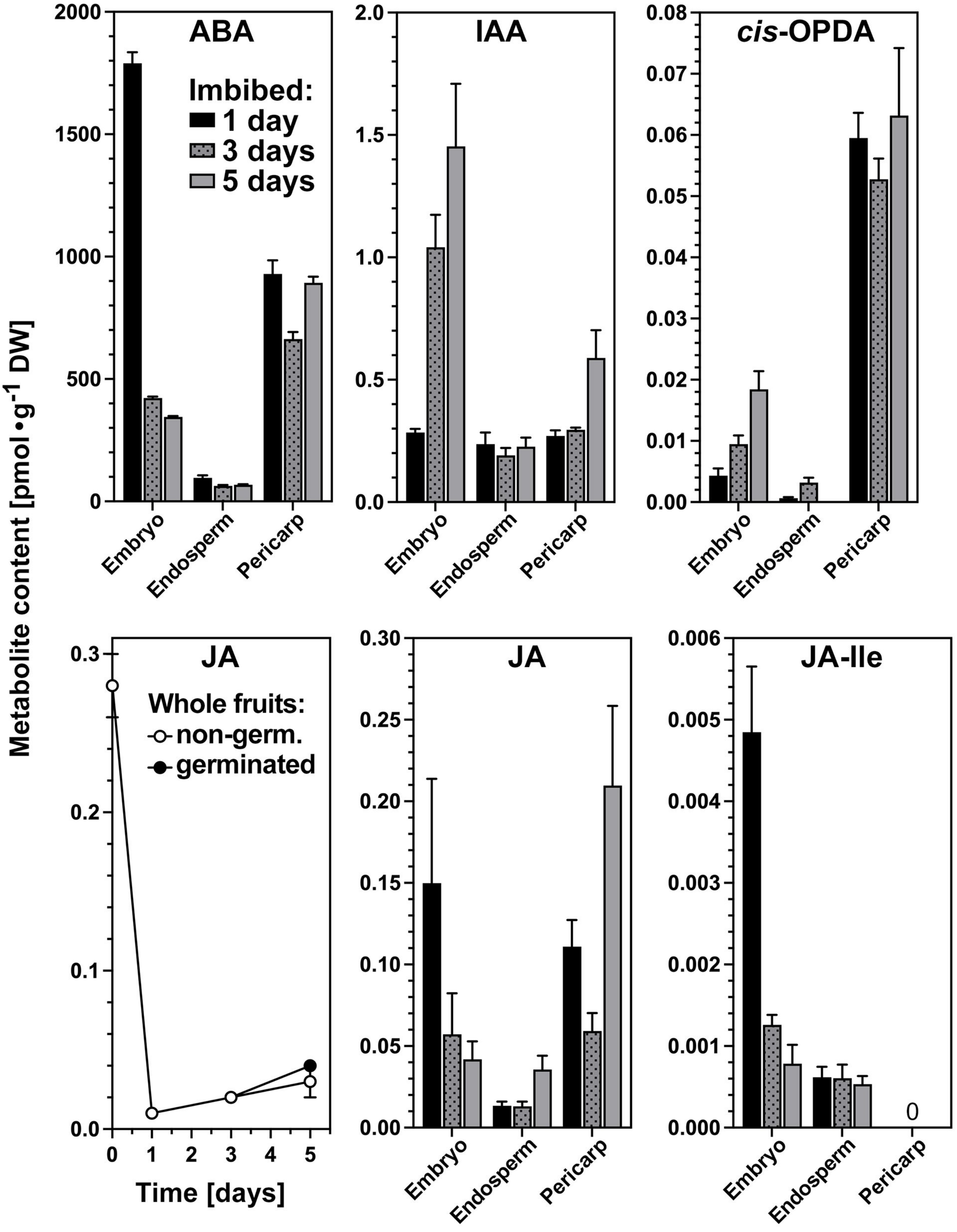

Supplementary Fig. S4. Spatiotemporal hormonal analysis of celery cultivar Victoria germination during imbibition at 20ºC in continuous white light. Metabolite contents per dry weight (DW) in dry and imbibed fruits and fruit compartments (embryo, endosperm, pericarp) at different times. ABA, abscisic acid, IAA, indole-3-acetic acid; JA, jasmonic acid; JA-Ile, jasmonoyl-L-isoleucine; *cis*-OPDA, *cis*-(+)-12-oxo-phytodienoic acid. Mean ± SEM values are presented obtained from five biological replicates with ~100 fruits used per replicate sample. Note that the hormone contents in celery fruit compartments differ considerably from parsnip fruit compartments (Figures 4 and S3) especially in the pericarp for which ABA, IAA and *cis*-OPDA were >3-fold, ~1,000-fold ~400,000,000-fold lower in celery when compared to parsnip dry fruits.

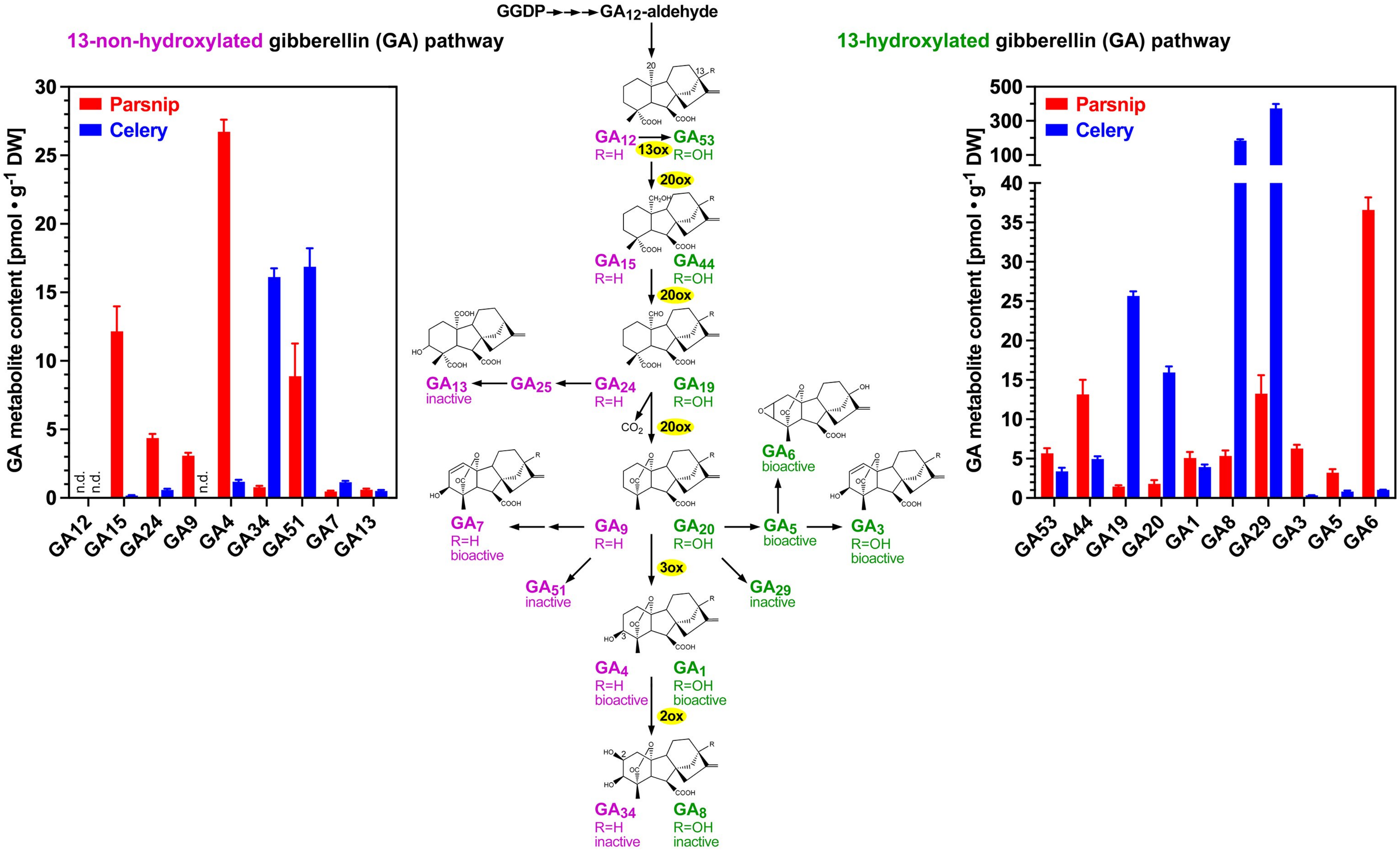

Supplementary Fig. S5. Gibberellin (GA) metabolite analysis of whole dry fruits of parsnip cultivar Panorama #3 and celery cultivar Victoria. Bioactive GA metabolites are indicated (Yamaguchi 2008); Metabolite contents per dry weight (DW); Mean ± SEM values are presented of 6 biological samples; n.d., not detected.

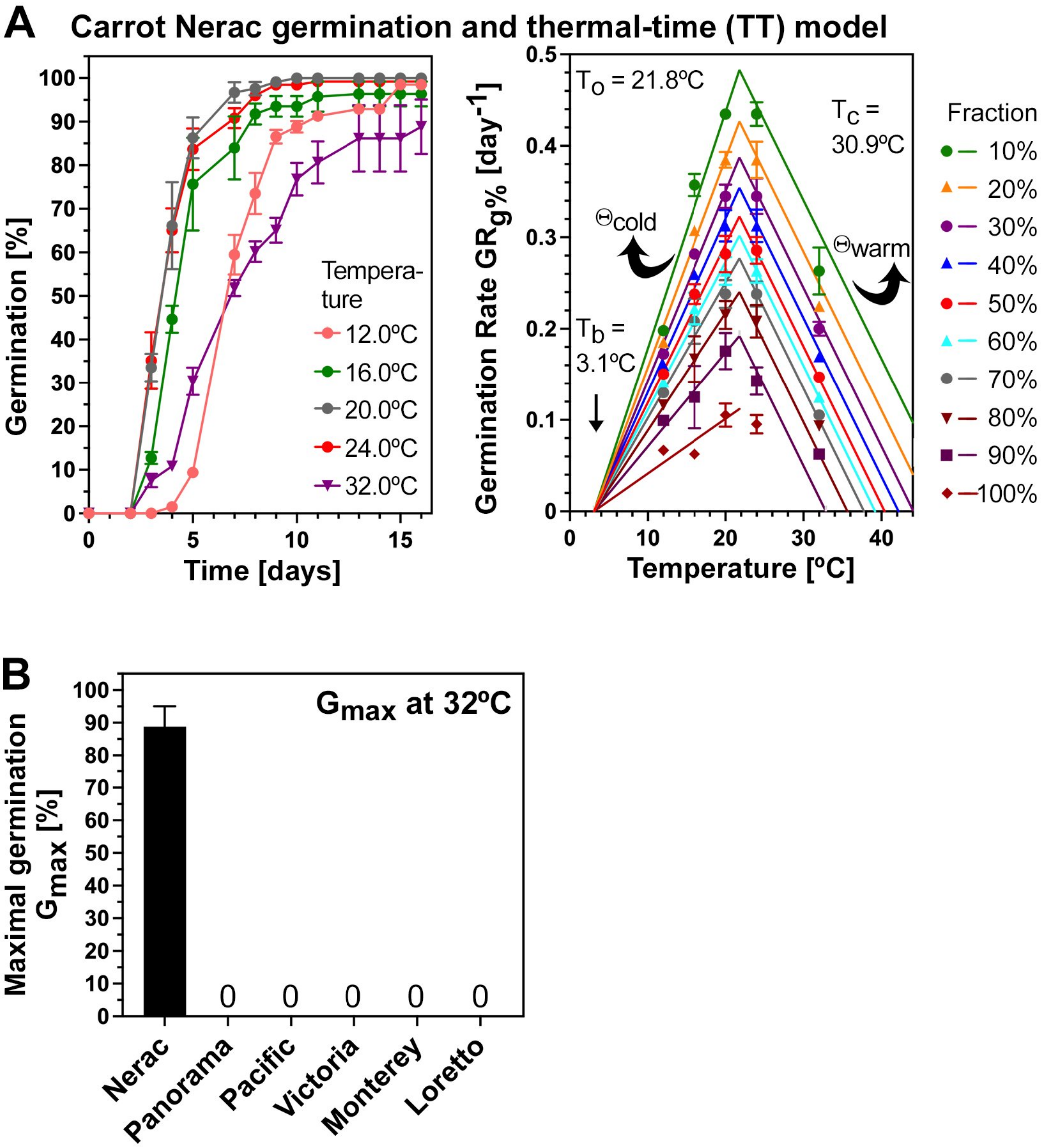

Supplementary Fig. S6. The effect of temperature mericarp germination of *Daucus carota* (carrot) and other Apiaceae. (A) Responses to different imbibition temperatures (*left panel*) and population-based thermal-time threshold modelling (*right panel*) of carrot cultivar Nerac germination in continuous white light. The thermal-time model delivered estimated cardinal temperatures T_b (base), T_o (optimal), T_c (ceiling or maximal) in ºC and the thermal-time constants Θ_{cold} and Θ_{warm} in ºC•days (derived fom the slopes of the regression lines; see the material and method section for details) are indicated in the figure and listed in Table 1. (B) Maximal germination percentages (G_{max}) of carrot (cultivar Nerac), parsnip (cultivars Panorama and Pacific) and celery (cultivars Victoria, Monterey, and Loretto) at 32ºC. Note that thermoinhibition blocked the germination of all parsnip and celery batches. In contrast to this, carrot cultivar Nerac germinated readily when imbibed at 32ºC. Mean ± SEM germination values presented are from triplicate plates each with 50 (celery, carrot) or 30 (parsnip) fruits.

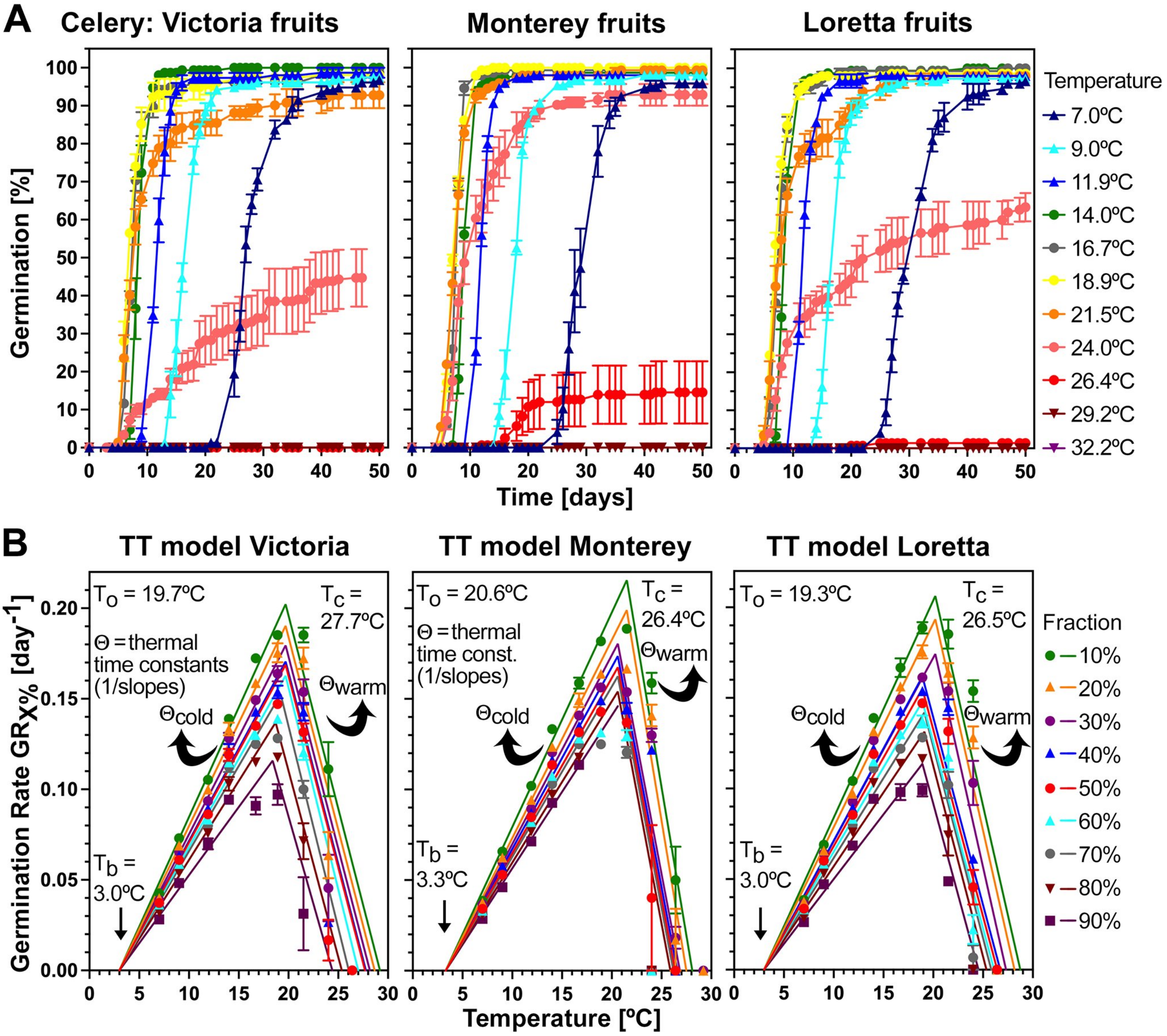

Supplementary Fig. S7. Comparative germination analysis of *Apium graveolens* temperature responses. (A) The effect of different imbibition temperatures on the germination of fruits of the three celery cultivars Victoria, Monterey and Loretta in continuous white light. Mean ± SEM values are presented for triplicate plates each with 50 fruits. (B) Population-based thermal-time (TT) threshold modelling of corresponding fruit and seed germination. The TT models delivered estimated cardinal temperatures T_b (base), T_o (optimal), T_c (ceiling or maximal) in ºC and the thermal-time constants Θ_{cold} and Θ_{warm} in ºC•days (derived fom the slopes of the regression lines; see the material and method section for details) as indicated in the figure and listed in Table 1.

Supplementary Table S1. Morphological mericarp (fruit) and seed properties of Apiaceae cultivars.

Species	Cultivar	Batch	Number	Grading [mm]	Mass TSW [g]	Entity	Length ± SEM [mm]
Parsnip	Panorama	#1	E64291	4.75-5.00	5.810	Fruit	5.581 ± 0.083
						Seed	4.514 ± 0.071
Parsnip	Panorama	#2	E57306	4.75-5.00	6.725	Fruit	5.623 ± 0.101
						Seed	4.572 ± 0.077
Parsnip	Pacific	#1	E64292	4.75-5.00	6.215	Fruit	6.095 ± 0.071
						Seed	4.821 ± 0.057
Parsnip	Pacific	#2	E57305	4.75-5.00	6.471	Fruit	5.979 ± 0.081
						Seed	4.805 ± 0.065
Parsnip	Panorama	#3	E38814	4.75-5.00	4.540	Fruit	4.900 ± 0.096
						Seed	4.095 ± 0.047
Parsnip	Panorama	#3	E38817	3.75-4.00	6.880	Fruit	6.079 ± 0.079
						Seed	3.397 ± 0.066
Celery	Victoria		03731094		0.310	Fruit	1.243 ± 0.011
						Seed	1.138 ± 0.011
Celery	Monterey		03731245		0.350	Fruit	1.168 ± 0.010
						Seed	1.061 ± 0.009
Celery	Loretta		037245382		0.280	Fruit	1.222 ± 0.011
						Seed	1.121 ± 0.011
Carrot	Nerac		E56490	1.6-1.8	1.007	Fruit	2.556 ± 0.040
						Seed	2.303 ± 0.037

Table S2. Relative embryo sizes of wild and cultivated Apiaceae species; EL, embryo length; E:F, embryo:fruit length ratio. The dataset is organised by phylogenetic groups A, B and others (Figure 2), type (crop, wild species, or landrace) and Apiaceae tribe (Walker et al. 2021). Original literature resources are provided; note that for original literature marked with "*", the data were obtained from the supplementary data of (Visscher et al. 2022).

Species name (and details)	Type	Group	Apiaceae tribe	Initial EL [mm]	Critical EL [mm]	Fruit length [mm]	Initial E:F ratio	Critical E:F ratio	Literature sources
Pastinaca sativa cv. Pacific #1	Crop	A	Tordylieae	1.67	3.45	6.10	0.274	0.567	This work
Pastinaca sativa cv. Pacific #2	Crop	A	Tordylieae	1.55	3.48	5.98	0.259	0.582	This work
Pastinaca sativa cv. Panorama #1	Crop	A	Tordylieae	1.45	3.00	5.58	0.259	0.538	This work
Pastinaca sativa cv. Panorama #2	Crop	A	Tordylieae	1.19	3.04	5.62	0.212	0.541	This work
Apium graveolens cv. Victoria	Crop	A	Apieae	0.32	1.01	1.24	0.255	0.812	(Walker et al. 2021)
Apium graveolens cv. Monterey	Crop	A	Apieae	0.30	0.95	1.17	0.255	0.813	This work
Apium graveolens cv. Loretta	Crop	A	Apieae	0.32	1.01	1.22	0.259	0.825	This work
Foeniculum vulgare cv. Fasa and cv. Chahestan [a]	Crop	A	Apieae	1.16	2.41	4.36	0.263	0.567	(Hashemirad et al. 2023)
Anethum graveolens cv. Kentavr [a]	Crop	A	Apieae	0.93	2.45	3.28	0.281	0.743	(Bukharov et al. 2021)
Anethum graveolens cv. Centaur [a]	Crop	A	Apiaae	0.85	2.45	3.35	0.260	0.730	(Soldatenko et al. 2020)
Hladnikia pastinacifolia	Wild	A	Careae	0.55	2.81	3.35	0.164	0.840	(Sajna et al. 2019)*
Aegopodium podagraria	Wild	A	Careae	0.35	2.86	2.96	0.138	0.965	(Vandelook et al. 2009)*
Sison amomum	Wild	A	Pyramidoptereae	2.10	4.92	6.00	0.350	0.820	(Van Assche et al. 2011)*
Cyclospermum leptophyllum	Wild	A	Pyramidoptereae	0.34	1.22	1.25	0.272	0.976	(Walck et al. 2008)*
Elwendia caroides	Wild	A	Pyramidoptereae	0.96	4.17	5.96	0.160	0.699	(Rahimi et al. 2024)
Elwendia wolfii	Wild	A	Pyramidoptereae	0.28	3.44	4.04	0.070	0.850	(Rahimi et al. 2024)
Bunium persicon (mean of four populations)	Wild	A	Pyramidoptereae				0.162	0.775	(Mohammadianfar et al. 2024)
Angelica keiskei	Wild	A	Selineae	2.31	9.07	10.67	0.216	0.850	(Zhang et al. 2019)*
Angelica sylvestris	Wild	A	Selineae	0.50	0.78	4.73	0.200	0.700	(Vandelook et al. 2007a)
Selinum carvifolia	Wild	A	Selineae	0.46	2.46	2.95	0.300	0.834	(Vandelook et al. 2007a)
Thaspium pinnatifidum	Wild	A	Selineae	0.70	3.60	4.00	0.175	0.900	(Baskin et al. 1992)*
Lomatium dissectum	Wild	A	Selineae	1.35	7.40	9.00	0.123	0.820	(Scholten et al. 2009)
Saposhnikovia divaricata	Wild	A	Selineae	0.96		5.66	0.171		(Yugrina et al. 2021)
Glehnia littoralis	Wild	A	Selineae	1.04		6.55	0.150		(Yeom et al. 2021)
Heracleum sphondylium	Wild	A	Tordylieae	1.00	4.00	5.01	0.094	0.798	(Stokes 1952)*
Pastinaca sativa (wild parsnip) [b]	Wild	A	Tordylieae	0.82	4.75		0.173		(Hendrix et al. 1991)*
Conium maculatum	Wild	A	*Conium* clade	0.90	1.70	2.77	0.325	0.614	(Baskin and Baskin 1990b)*
Prangos ferulacea	Wild	A	*Cachrys* clade	3.50	7.00	12.00	0.292	0.583	(Razavi and Hajiboland 2009)*

Species name (and details)	Type	Group	Apiaceae tribe	Initial EL [mm]	Critical EL [mm]	Fruit length [mm]	Initial E:F ratio	Critical E:F ratio	Source
Daucus carota cv. Nerac	Crop	B	Daucinae	0.85	2.00	2.56	0.334	0.786	This work
Daucus carota cv. Chanteney [c]	Crop	B	Daucinae	1.34		3.37	0.401		(Gray et al. 1988)
Cuminum cyminum (mean of 15 Iranian and Indian landraces)	Landr.	B	Daucinae	1.42		5.16	0.274		(Soltani et al. 2019)
Daucus carota (mean of 16 wild carrot accessions from Europe)	Wild	B	Daucinae				0.328		(Vandelook et al. 2024)
Daucus carota (mean of 24 wild carrot accessions from North America)	Wild	B	Daucinae				0.249		(Vandelook et al. 2024)
Pterygopleurum neurophyllum	Wild	B	*Acronema* clade	0.23	0.99	2.42	0.096	0.411	(Kwon et al. 2020)*
Smyrnium cordifolium	Wild	B	Smyrnieeae				0.200	0.820	(Zarei-Gavkosh et al. 2022)
Bubon macedonicum	Wild	B	Daucinae	0.60	1.67	1.81	0.334	0.924	(Di Cecco et al. 2018)*
Ferula gummosa	Wild	B	Ferulinae	1.70	3.35	5.05	0.337	0.663	(Zardari et al. 2019)*
Ferula ovina	Wild	B	Ferulinae	3.33	8.19	9.00	0.370	0.910	(Fasih and Afshari 2018)*
Osmorhiza longistylis	Wild	B	Scandicinae	0.60	8.80	13.40	0.062	0.657	(Baskin et al. 1995)*
Osmorhiza aristata	Wild	B	Scandicinae	0.48	9.00	11.10	0.043	0.811	(Walck et al. 2002)*
Osmorhiza depauperata	Wild	B	Scandicinae	0.58	9.00	9.62	0.060	0.936	(Walck and Hidayati 2004)*
Osmorhiza occidentalis	Wild	B	Scandicinae	1.22	9.40	10.00	0.122	0.940	(Baskin et al. 1995)*
Osmorhiza aristata var. *aristata*	Wild	B	Scandicinae	0.50	9.80	10.30	0.049	0.951	(Baskin and Baskin 1991)*
Osmorhiza chilensis	Wild	B	Scandicinae	0.60	9.20	9.50	0.063	0.968	(Baskin et al. 1995)*
Chaerophyllum aureum	Wild	B	Scandicinae	1.07	4.80	8.03	0.133	0.598	(Santiago et al. 2019)*
Chaerophyllum tainturieri	Wild	B	Scandicinae	0.47	2.80	4.00	0.107	0.700	(Baskin and Baskin 1990a)*
Chaerophyllum bulbosum	Wild	B	Scandicinae	0.50	3.55	4.64	0.108	0.765	(Janiesch 1971)*
Chaerophyllum procumbens	Wild	B	Scandicinae	0.50	4.60	5.00	0.100	0.920	(Baskin and Baskin 1990a)*
Chaerophyllum temulum	Wild	B	Scandicinae	0.53	3.24	3.41	0.156	0.950	(Vandelook et al. 2007b)
Anthriscus sylvestris	Wild	B	Scandicinae	0.45	4.57	5.00	0.090	0.914	(Baskin et al. 2000)*
Conopodium majus	Wild	B	Scandicinae	0.30	2.09	2.22	0.135	0.941	(Blandino et al. 2019)*
Torilis japonica	Wild	B	Torilidinae	0.25	0.93	2.25	0.111	0.413	(Vandelook et al. 2008)*
Torilis scabra	Wild	B	Torilidinae	1.10	3.30	4.78	0.230	0.690	(Zhang et al. 2023)
Turgenia latifolia	Wild	B	Torilidinae	3.99	8.30	10.51	0.380	0.790	(Nurulla et al. 2014)*

Species name (and details)	Type	Group	Apiaceae tribe	Initial EL [mm]	Critical EL [mm]	Fruit length [mm]	Initial E:F ratio	Critical E:F ratio	Source
Cicuta virosa	Wild	Other	Oenantheae	0.33	0.92	1.95	0.170	0.469	(Cho et al. 2018)*
Trepocarpus aethusae	Wild	Other	Oenantheae	1.81	4.16	8.00	0.064	0.520	(Baskin et al. 2003)*
Cryptotaenia canadensis	Wild	Other	Oenantheae	1.60	3.00	4.81	0.333	0.624	(Baskin and Baskin 1988)*
Perideridia americana	Wild	Other	Oenantheae	0.30	2.80	3.00	0.100	0.933	(Baskin and Baskin 1993)*
Ptilimnium nutallii	Wild	Other	Oenantheae	0.22	1.25	1.26	0.175	0.992	(Baskin et al. 1999)*
Bupleurum kakiskalae	Wild	Other	Bupleureae	0.57	1.51	1.89	0.300	0.800	(Visscher et al. 2022)
Sanicula trifoliata	Wild	Other	Saniculeae	0.50	2.99	6.00	0.083	0.498	(Hawkins et al. 2010)*
Sanicula canadensis	Wild	Other	Saniculeae	0.36	1.58	2.00	0.123	0.790	(Hawkins et al. 2010)*
Sanicula gregaria	Wild	Other	Saniculeae	0.38	2.54	3.00	0.127	0.847	(Hawkins et al. 2010)*
Sanicula europaea	Wild	Other	Saniculeae	0.32	2.10	2.38	0.136	0.883	(Vandelook and Van Assche 2008)*
Eryngium sparanophyllum	Wild	Other	Saniculeae	0.55	1.51	2.42	0.236	0.602	(Wolkis et al. 2020)
Eryngium yuccifolium	Wild	Other	Saniculeae	0.98	2.86	3.01	0.326	0.950	(Schutte and Knee 2005)*
Eryngium viviparum	Wild	Other	Saniculeae	0.42	1.00	1.03	0.310	0.978	(Ayuso et al. 2017)

[a] To calculate seed length (S) from the fruit length results in the table, and from there E:S ratios presented in Figure 8D, the thickness of the pericarp data for these species published by Ma *et al.* (2015) was used. For *Anethum graveolens* mean ±SEM of primary and secondary umbels, and for *Foeniculum vulgare* mean ±SEM of two cultivars are presented.

[b] Fruit length was calculated from the fruit mass data presented in Figure 1 of Hendrix et al. (1991) based on a linear relationship (R^2 = 0.74) between fruit mass and fruit length data of cultivated parsnip fruits (Supplementary Table S1); the E:F ratio is the mean ± SEM (0.173 ± 0.004) calculated from 72 datapoints for wild parnip collected from a pasture in Iowa (Hendrix et al. 1991).

[c] Fruit length was calculated from the fruit mass data presented in Table 2 of Gray et al. (1988) based on a linear relationship (R^2 = 0.92) between fruit mass and fruit length data of carrot cultivar Nerac (Supplementary Table S1) plus data from 10 wild carrot accessions fruits (Kadluczka and Grzebelus 2022); results are supported by several publications by Gray et al. (1988; 1984) on this carrot cultivar.

References for Supplementary Table S2

Ayuso M, Ramil-Rego P, Landin M, Gallego PP, Barreal ME (2017) Computer-assisted recovery of threatened plants: keys for breaking seed dormancy of *Eryngium viviparum*. Front Plant Sci 8:2092. doi:10.3389/fpls.2017.02092

Baskin CC, Baskin JM, Chester EW (1999) Seed dormancy in the wetland winter annual *Ptilimnium nuttallii* (Apiaceae). Wetlands 19:359-364. doi:10.1007/Bf03161767

Baskin CC, Baskin JM, Chester EW (2003) Ecological life cycle of *Trepocarpus aethusae* (Nutt.) ex DC. and comparisons with two other winter annual Apiaceae native to Easthern United States. Castanea 68:43-55. doi:https://www.jstor.org/stable/4034156

Baskin CC, Chester EW, Baskin JM (1992) Deep complex morphophysiological dormancy in seeds of *Thaspium pinnatifidum* (Apiaceae). International Journal of Plant Sciences 153:565-571. doi:10.1086/297080

Baskin CC, Meyer SE, Baskin JM (1995) Two types of morphophysiological dormancy in seeds of two genera (*Osmorhiza* and *Erythronium*) with an Arcto-Tertiary distribution pattern. Am J Bot 82:293-298. doi:10.2307/2445574

Baskin CC, Milberg P, Andersson L, Baskin JM (2000) Deep complex morphophysiological dormancy in seeds of *Anthriscus sylvestris* (Apiaceae). Flora 195:245-251. doi:10.1016/S0367-2530(17)30977-5

Baskin JM, Baskin C (1988) The ecological life cycle of *Cryptotaenia canadensis* (L.) DC. (Umbelliferae), a woodland herb with monocarpic ramets. The American Midland Naturalist 119:165-173. doi:10.2307/2426065

Baskin JM, Baskin C (1993) The ecological life cycle of *Perideridia americana* (Apiaceae). The American Midland Naturalist 129:76-86. doi:10.2307/2426437

Baskin JM, Baskin CC (1990a) Germination ecophysiology of seeds of the winter annual *Chaerophyllum tainturieri* - a new type of morphophysiological dormancy. J Ecol 78:993-1004. doi:10.2307/2260948

Baskin JM, Baskin CC (1990b) Seed germination ecology of poison hemlock, *Conium maculatum*. Can J Bot 68:2018-2024. doi:10.1139/b90-264

Baskin JM, Baskin CC (1991) Nondeep complex morphophysiological dormancy in seeds of *Osmorhiza claytonii* (Apiaceae). Am J Bot 78:588-593. doi:10.2307/2445268

Blandino C, Fernandez-Pascual E, Marin M, Vernet A, Pritchard HW (2019) Seed ecology of the geophyte *Conopodium majus* (Apiaceae), indicator species of ancient woodland understories and oligotrophic meadows. Plant Biol 21:487-497. doi:10.1111/plb.12872

Bukharov AF, Baleev DN, Soldatenko AV, Musaev FB, Kezimana P, Priyatkin NS (2021) Impacts of high temperature on embryonic growth and seed germination of dill (*Anethum graveolens*). Seed Sci Technol 49:7-17. doi:10.15258/sst.2021.49.1.02

Cho JS, Jang BK, Lee CH (2018) Seed dormancy and germination characteristics of the endangered species *Cicuta virosa* L. in South Korea. Hortic Environ Biote 59:473-481. doi:10.1007/s13580-018-0062-7

Di Cecco V, Paura B, Bufano A, Di Santo P, Di Martino L, Frattaroli AR (2018) Analysis of diaspore morphology and seed germination in *Bubon macedonicum* L., a rare species in Italy. Plant Biosyst 152:738-748. doi:10.1080/11263504.2017.1330775

Fasih M, Afshari RT (2018) The morphophysiological dormancy of *Ferula ovina* seeds is alleviated by low temperature and hydrogen peroxide. Seed Sci Res 28:52-62. doi:10.1017/S0960258517000356

Gray D, Steckel JRA, Dearman J, Brocklehurst PA (1988) Some effects of temperature during seed development on carrot (*Daucus carota)* seed gowth and quality. Annals of Applied Biology 112:367-376. doi:10.1111/j.1744-7348.1988.tb02073.x

Gray D, Ward JA, Steckel JRA (1984) Endosperm and embryo development in *Daucus carota* L. J Exptl Bot 35:459-465. doi:10.1093/jxb/35.4.459

Hashemirad S, Soltani E, Darbandi AI, Alahdadi I (2023) Cold stratification requirement to break morphophysiological dormancy of fennel (*Foeniculum vulgare* Mill.) seeds varies with seed length. J Appl Res Med Aroma 35:100465. doi:10.1016/j.jarmap.2023.100465

Hawkins TS, Baskin CC, Baskin JM (2010) Morphophysiological dormancy in seeds of three eastern North American *Sanicula* species (Apiaceae subf. Saniculoideae): evolutionary implications for dormancy break. Plant Species Biology 25:103-113. doi:10.1111/j.1442-1984.2010.00273.x

Hendrix SD, Nielsen E, Nielsen T, Schutt M (1991) Are seedlings from small seeds always inferior to seedlings from large seeds? Effects of seed biomass on seedling growth in *Pastinaca sativa* L. New Phytol 119:299-305. doi:10.1111/j.1469-8137.1991.tb01034.x

Janiesch P (1971) On the physiology of the after-ripening of Umbelliferae from nitrophilous hedge-banks. Flora 160:518-525. doi:10.1016/S0367-2530(17)32033-9

Kadluczka D, Grzebelus E (2022) Comparative fruit morphology and anatomy of wild relatives of carrot (*Daucus*, Apiaceae). Agriculture 12:2104. doi:10.3390/agriculture12122104

Kwon HJ, Kim YR, Kim SY (2020) First report on the germination requirement of *Pterygopleurum neurophyllum* seeds. Seed Sci Technol 48:41-48. doi:10.15258/sst.2020.48.1.06

Ma XD, Mao WW, Zhou P, Li P, Li HJ (2015) Distinguishing *Foeniculum vulgare* fruit from two adulterants by combination of microscopy and GC-MS analysis. Microscopy Research and Technique 78:633-641. doi:10.1002/jemt.22523

Mohammadianfar E, Ghaderi-Far F, Torabi B, Siahmarguee A, Behroj M, Baskin C, Sadeghipour HR (2024) Variation in degree of intermediate complex morphophysiological dormancy in seeds of *Bunium persicum* (Apiaceae). Plant Species Biology 39:3-13. doi:10.1111/1442-1984.12436

Nurulla M, Baskin CC, Lu JJ, Tan DY, Baskin JM (2014) Intermediate morphophysiological dormancy allows for life-cycle diversity in the annual weed, *Turgenia latifolia* (Apiaceae). Aust J Bot 62:630-637. doi:10.1071/Bt14281

Rahimi H, Rezanejad F, Ayatollahi SA, Sharifi-Sirchi GR, Rahimi H (2024) Embryo growth and seed germination requirements in underdeveloped embryos of *Elwendia caroides* and *E. wolfii* (Apiaceae). Mediterr Bot 45:e91058. doi:10.5209/mbot.91058

Razavi SM, Hajiboland R (2009) Dormancy breaking and germination of *Prangos ferulaceae* seeds. Eurasian Journal of Bioscience 3:78-83. doi:10.5053/ejobios.2009.3.0.11

Sajna N, Sipek M, Sustar-Vozlic J, Kaligaric M (2019) Germination behavior of the extremely rare *Hladnikia pastinacifolia* Rchb. (Apiaceae) - a Pleistocene in situ survivor. Acta Bot Croat 78:107-115. doi:10.2478/botcro-2019-0017

Santiago A, Ahrazem O, Gómez-Gómez L, Copete MA, Herranz R, Ferrandis P (2019) Seed germination requirements of relictic and broadly-distributed populations of *Chaerolhyllum aureum* (Apiaceae): connecting ecophysiology and genetic identity. Turkish Journal of Botany 43:320-330. doi:10.3906/bot-1807-42

Scholten M, Donahue J, Shaw NL, Serpe MD (2009) Environmental regulation of dormancy loss in seeds of *Lomatium dissectum* (Apiaceae). Ann Bot 103:1091-1101. doi:10.1093/aob/mcp038

Schutte B, Knee M (2005) The effects of rudimentary embryos and elevated oxygen on seed dormancy of *Eryngium yuccifolium* Michx. (Apiaceae). Seed Sci Technol 33:53-62. doi:10.15258/sst.2005.33.1.06

Soldatenko AV, Buharov AF, Baloov DN, Ivanova MI, Nazarov PA, Razin OA, Razin AF (2020) Impact of high temperature on growth of vembryo and germination of heteromorphic seeds os *Anethum graveolens* L. (Apiaceae). Agricultural Biology 55:932-944. doi:10.15398/agrobiology.2020.5.932eng

Soltani E, Mortazavian SMM, Faghihi S, Akbari GA (2019) Non-deep simple morphophysiological dormancy in seeds of *Cuminum cyminum* L. J Appl Res Med Aroma 15:100222. doi:10.1016/j.jarmap.2019.100222

Stokes P (1952) A physiological study of embryo development in *Heracleum sphondylium* L.: I. The effect of temperature on embryo development. Ann Bot 16:441-447. doi:https://doi.org/10.1093/oxfordjournals.aob.a083326

Van Assche JA, Cornelissen D, Vandelook F (2011) Germination ecology of *Sison amomum* (Apiaceae) at the northern edge of its distribution range on the European mainland. Plant Ecol Evol 144:321-326. doi:10.5091/plecevo.2011.638

Vandelook F, Bolle N, Van Assche JA (2007a) Multiple environmental signals required for embryo growth and germination of seeds of *Selinum carvifolia* (L.) L. and *Angelica sylvestris* L. (Apiaceae). Seed Sci Res 17:283-291. doi:10.1017/S0960258507838888

Vandelook F, Bolle N, Van Assche JA (2007b) Seed dormancy and germination of the European *Chaerophyllum temulum* (Apiaceae), a member of a trans-Atlantic genus. Ann Bot 100:233-239. doi:10.1093/aob/mcm090

Vandelook F, Bolle N, Van Assche JA (2008) Seasonal dormancy cycles in the biennial *Torilis japonica* (Apiaceae), a species with morphophysiological dormancy. Seed Sci Res 18:161-171. doi:10.1017/S0960258508038877

Vandelook F, Bolle N, Van Assche JA (2009) Morphological and physiological dormancy in seeds of *Aegopodium podagraria* (Apiaceae) broken successively during cold stratification. Seed Sci Res 19:115-123. doi:10.1017/S0960258509301075

Vandelook F, Van Assche JA (2008) Deep complex morphophysiological dormancy in *Sanicula europaea* (Apiaceae) fits a recurring pattern of dormancy types in genera with an Arcto-Tertiary distribution. Botany-Botanique 86:1370-1377. doi:10.1139/B08-103

Vandelook F, van de Vyver A, Soltani E (2024) Intra-specific variation in relative embryo length and germination of wild *Daucus carota* across climate gradients in North America and Europe. Seed Sci Res 34:207-217. doi:10.1017/S0960258524000230

Visscher AM, Vandelook F, Fernandez-Pascual E, Perez-Martinez LV, Ulian T, Diazgranados M, Mattana E (2022) Low availability of functional seed trait data from the tropics could negatively affect global macroecological studies, predictive models and plant conservation. Ann Bot 130:773-784. doi:10.1093/aob/mcac130

Walck JL, Baskin CC, Hidayati SN, Baskin JM (2008) Comparison of the seed germination of native and non-native winter annual Apiaceae in North America, with particular focus on *Cyclospermum leptophyllum* naturalized from South America. Plant Species Biology 23:33-42. doi:10.1111/j.1442-1984.2008.00205.x

Walck JL, Hidayati SN (2004) Germination ecophysiology of the western North American species *Osmorhiza depauperata* (Apiaceae): implications of preadaptation and phylogenetic niche conservatism in seed dormancy evolution. Seed Sci Res 14:387-394. doi:10.1079/Ssr2004184

Walck JL, Hidayati SN, Okagami N (2002) Seed germination ecophysiology of the Asian species *Osmorhiza aristata* (Apiaceae): Comparison with its North American congeners and implications for evolution of types of dormancy. Am J Bot 89:829-835. doi:10.3732/ajb.89.5.829

Walker M, Pérez M, Steinbrecher T, Gawthrop F, Pavlovic I, Novák O, Tarkowská D, Strnad M, Marone F, Nakabayashi K, Leubner-Metzger G (2021) Molecular mechanisms and hormonal regulation underpinning morphological dormancy: a case study using *Apium graveolens* (Apiaceae). Plant J 108:1020-1036. doi:10.1111/tpj.15489

Wolkis D, Blackwell S, Villanueva SK (2020) Conservation seed physiology of the cienega endemic, *Eryngium sparganophyllum* (Apiaceae). Conserv Physiol 8:coaa017. doi:10.1093/conphys/coaa017

Yamaguchi S (2008) Gibberellin metabolism and its regulation. Annual Review of Plant Biology 59:225-251. doi:10.1146/annurev.arplant.59.032607.092804

Yeom MS, Nguyen TKL, Cho JS, Oh MM (2021) Improving germination rate of coastal *Glehnia* by cold stratification and pericarp removal. Agronomy 11:944. doi:10.3390/agronomy11050944

Yugrina P, Urbagarova B, Elisafenko T (2021) Morphological features of fruits and seeds of *Saposhnikovia divaricata* (Apiaceae). BIO Web of Conferences 38:00141. doi:10.1051/bioconf/20213800141

Zardari S, Ghaderi-Far F, Sadeghipour HR, Zeinali E, Soltani E, Baskin CC (2019) Deep and intermediate complex morphophysiological dormancy in seeds of *Ferula gummosa* (Apiaceae). Plant Species Biology 34:85-94. doi:10.1111/1442-1984.12238

Zarei-Gavkosh M, Afshari RT, Jahansooz M (2022) Morphophysiological dormancy in *Smyrnium cordifolium* Boiss: Germination requirements and embryo growth. J Appl Res Med Aroma 30:100385. doi:10.1016/j.jarmap.2022.100385

Zhang KL, Zhang Y, Walck JL, Tao J (2019) Non-deep simple morphophysiological dormancy in seeds of *Angelica keiskei* (Apiaceae). Sci Hortic-Amsterdam 255:202-208. doi:10.1016/j.scienta.2019.05.039

Zhang L, Xu CH, Liu HN, Tao J, Zhang KL (2023) Seed dormancy and germination requirements of *Torilis scabra* (Apiaceae). Agronomy 13:1250. doi:10.3390/agronomy13051250
